# Composite Hydrogel Loading Polysaccharides Derived from *Coptis chinensis* Franch. for Promoting Diabetic Wound Healing

**DOI:** 10.3390/biom16081162

**Published:** 2026-08-10

**Authors:** Menghan Li, Bin Zhang, Youyan Zeng, Yongxin Mao, Jinyi Zhang, Tingfang Zhao, Huanglin Huo, Huicong Zeng, Qian Zhou, Bo Li

**Affiliations:** 1 Key Laboratory of Glucolipid Metabolic Diseases, Ministry of Education, Guangdong Pharmaceutical University, Guangzhou 510006, China; 2 Guangdong Provincial TCM Key Laboratory for Metabolic Diseases, Guangdong Pharmaceutical University, Guangzhou 510006, China; 3 Traditional Chinese Medicine Research Institute, Guangdong Pharmaceutical University, Guangzhou 510006, China; 4 Guangdong Provincial Research Center of Integration of Traditional Chinese Medicine and Western Medicine in Metabolic Diseases, Guangdong Pharmaceutical University, Guangzhou 510006, China; 5 Key Laboratory of Tumor Molecular Biology, Ministry of Education, College of Life Science and Technology, Jinan University, Guangzhou 510632, China; 6 Key Laboratory of Functional Protein Research of Guangdong Higher Education Institutes, College of Life Science and Technology, Jinan University, Guangzhou 510632, China

**Keywords:** diabetic wound healing, *Coptis chinensis*, polysaccharide, composite hydrogels

## Abstract

Efficient treatment of diabetic wounds (DW) remains a major clinical challenge worldwide owing to vascular insufficiency, multiple bacterial infections, and overactivation of pro-inflammatory M1 macrophages caused by hyperglycemia. The development of novel pharmaceutical agents with multiple biological functions is urgently needed. *Coptis chinensis* Franch. (CC) has been used to treat diabetes for thousands of years in China, but the curative effects and underlying mechanisms of CC in DW remain uncertain. Herein, a homogeneous heteropolysaccharide component, namely CCP, was isolated and purified from CC, which exhibited a molecular weight of 39,697 Da and was primarily composed of Glc, GalA, Ara, Gal, and Xyl. CCP has a light yellowish color and is distributed in a block shape with small surface granulations. In vitro experiments revealed that CCP dose-dependently mitigated high glucose-induced suppression of viability, migration, and tube formation in HUVECs. Meanwhile, CCP promotes the polarization of M1 macrophages toward the M2 phenotype to exert anti-inflammatory effects, while possessing certain antibacterial properties. In addition, a composite hydrogel system was successfully constructed by introducing sodium carboxymethyl cellulose and carbomer 940 for CCP delivery. The obtained hydrogels exhibited reasonable moisturizing, swelling, and drug release capacities, along with favorable rheological behaviors and certain antibacterial activity. More importantly, the in vivo wound healing model evaluation in diabetic rats demonstrated that CCP hydrogel dressings could effectively promote wound healing by reducing inflammation, accelerating collagen deposition, upregulating the expression of VEGF and key angiogenesis-related factors. In addition, composite hydrogels demonstrated excellent cytocompatibility and hemocompatibility, which holds great promise for clinical application in DW treatment.

## 1. Introduction

Diabetes mellitus constitutes a major public health problem worldwide, and diabetic wounds (DW) are among the most complex, costly, and frequent complications in patients with diabetes mellitus [1]. By 2030, the number of diabetics worldwide will reach 552 million, among whom 15–25% will develop DW in their lifetime, and some individuals may even subsequently progress to recurrent ulcers, which can lead to amputation [2,3]. Compared with normal wounds, the hyperglycemic environment of DW is more favorable to bacterial proliferation and accumulation, which further leads to persistent bacterial infections and chronic inflammation. Hyperglycemia can also damage endothelial cells, disrupting their proliferative, angiogenesis, and migratory functions [4]. Moreover, the high-glucose pathological environment increases oxidative stress via multiple mechanisms, including the direct generation of ROS or altering the redox balance [5]. This persistent oxidative stress further aggravates inflammatory cell infiltration and activates inflammatory signaling pathways, which induce a sustained release of inflammatory factors and amplify the inflammatory cascade [6]. This chronic inflammatory microenvironment of DW not only directly impairs the functions of keratinocytes and fibroblasts but also delays the transition from the inflammatory phase to the proliferative and remodeling phase, thereby causing DW healing failure [7]. Current clinical treatment methods for DW include antibiotics, growth factors, glycemic control, surgical debridement, and wound dressings, all of which have certain clinical efficacy and application value [8]. However, there is still limited efficacy of those single therapies, and multi-pathway synergistic therapy has been implicated as a key to effective DW treatment [9].

Traditional Chinese medicine has attracted increasing attention for the treatment of DW because of its multi-component, multi-target, multi-mechanism, overall regulation, and low side effects [10]. *Coptis chinensis* (CC), commonly known as “Huang Lian” in Chinese, is a well-recognized traditional Chinese herb that has been widely used to treat diabetes and its complications for thousands of years [11]. CC was first mentioned in Shen Nong’s Classic of Materia Medica and described as clearing heat, drying dampness, purging fire, and detoxifying [12]. Modern pharmacological research illustrates that CC has multiple properties, including antibacterial, anti-inflammatory, antioxidant, anti-tumor, antidiabetic, hypoglycemic, and immunomodulatory [13]. These extensive bioactivities have been ascribed to the presence of multiple components, such as berberine, coptisine, alkaloids, and polysaccharides [14]. However, the efficacy and potential mechanisms of CC in DW healing have not been fully elucidated. Recently, medicinal plant polysaccharides have received increasing attention due to their various biological activities and low toxicity [15]. A series of studies have shown that the polysaccharides from CC (CCP) possess diverse biological effects. Jiang et al. employed the glucose oxidase method to demonstrate that CCP enhances antioxidant activity by boosting the activity of superoxide dismutase and catalase, thereby mitigating ROS-induced DNA damage [16]. Using a diabetic rat model, Jiang et al. provided in vivo evidence that CCP exerts a robust inhibitory effect on the JAK2/STAT3 inflammatory signaling pathway, thereby mitigating excessive inflammatory responses in diabetic animals [17]. Moreover, CCP has also been shown to possess antibacterial activity [18]. These findings suggest that CCP may have potential as a therapeutic agent for DW treatment.

Traditional wound dressings, including gauze, absorbent cotton, and bandages, have been widely used by clinicians, but their poor moisturizing effect and lack of biological properties often lead to adhesions and hinder the healing trajectory [19]. Recently, inorganic nanomaterials, typified by metallic nanozymes, have attracted extensive attention for their applications in anti-infection and wound healing, owing to their unique properties, including photothermal, photodynamic, sonodynamic, and peroxidase-mimetic behaviors [20,21]. However, most inorganic nanomaterials lack a three-dimensional scaffold structure and tissue adhesiveness, failing to adapt to irregular wound interfaces. Additionally, their limited water retention impedes a favorable moist wound microenvironment. In comparison, composite hydrogels have attracted attention in wound healing because of their 3D porous structures with excellent plasticity, granting them the facilitator of drug release and wound exudate absorption [22,23]. Among them, composite hydrogels formed from polysaccharides in traditional Chinese medicine have been recognized as one of the most ideal wound dressing materials [24]. Zhang et al. prepared a novel hydrogel through cross-linking Aloe polysaccharide, honey, and PVA with borax, which showed excellent physicochemical properties and could accelerate skin wound healing [25]. An innovative composite hydrogel based on gelatin/oxidized carboxymethyl cellulose with fucoidan derived from Ecklonia cava has also proven effective in promoting full-thickness wound healing due to its sustained drug release ability [26]. Recently, cellulose-based hydrogels were fabricated through multiple hydrogen bonds cross-linking between sodium carboxymethylcellulose (CMC-Na) and Carbomer 940 (CBM940), as the ideal type of wound dressing, which has great potential for application to healing chronic wounds due to its excellent hydrophilicity, biocompatibility, and adhesiveness [27]. Previous studies suggested that *Bletilla striata* polysaccharide-loaded CMC-Na/CBM940 hydrogel combined the advantageous bioactivities of the individual components and showed significant wound healing-promoting ability [28]. Our previous results show that the polysaccharide of *Atractylodes macrocephala Koidz.* is capable of forming a hydrogel with CMC-Na/CBM940 through hydrogen bonds and physical entanglements, and this composite hydrogel can significantly improve diabetic wound healing by the synergistic action of the different mechanisms [29].

Herein, CCP was successfully isolated and purified from CC via water decoction and the Sevag method. The monosaccharide composition, molecular weight, surface morphology, physical and chemical properties of CCP were further evaluated using various characterization methods. Next, a high-glucose (HG) induced human umbilical vein endothelial cells (HUVECs) model was established to investigate its ability to protect and promote functions in HUVECs proliferation, migration, and tube formation. The effects of CCP on proinflammatory and anti-inflammatory factors were also investigated in the RAW264.7 macrophage inflammatory model. Meanwhile, the antibacterial activities of CCP against *Escherichia coli* (*E. coli*) and *Staphylococcus aureus* (*S. aureus*) were examined using agar plate colony counting. Afterward, a novel composite hydrogel was constructed by embedding CCP into CMC-Na/CBM940 to fill the wound bed and promote topical therapy effects. The antibacterial activity and physicochemical properties of the obtained hydrogels, including morphological characteristics, drug release, swelling ratio, degradation rate, adhesion, and rheological behavior, were comprehensively evaluated. Finally, the blood biocompatibility and cytotoxicity of the prepared hydrogels were assessed, and its wound healing efficacy and potential mechanisms were further validated in a full-thickness excisional diabetic rat model.

## 2. Materials and Methods

### 2.1. Materials

CC was purchased from Beijing Kangmei Pharmaceutical Co., Ltd. (Beijing, China). Jingwanhong ointment (JWH) was obtained from Tianjin Darentang Jingwanhong Pharmaceutical Co., Ltd. (Tianjin, China). D-glucose, bovine serum albumin, CMC-Na, CBM940, triethanolamine were purchased from Shanghai Aladdin Biochemical Technology Co., Ltd. (Shanghai, China). Dulbecco’s modified Eagle’s medium (DMEM), penicillin, fetal bovine serum (FBS), and streptomycin were purchased from Gibco (Waltham, MA, USA). Cell counting kit-8 (CCK-8) was purchased from Selleck (Houston, TX, USA). *S. aureus* and *E. coli* were purchased from Mingzhou Bio (Ningbo, China). Lysogeny broth (LB) and LB agar were purchased from Beyotime (Hangzhou, China). STAT3, phosphor-STAT3, and β-actin antibodies were purchased from Immunoway (San Jose, CA, USA); HRP-labeled secondary antibody was purchased from Absin (Shanghai, China). The 4% paraformaldehyde, HE and Masson stain, vWF, TGF-β1, CD31, VEGF, TNF-α, IL-6, and IL-10 antibodies were purchased from Wuhan Servicebio Technology Co., Ltd. (Wuhan, China).

### 2.2. Extraction and Purification of CCP

The CC powder (10 g) was mixed with distilled water (250 mL) and heated to reflux. The decoction was collected and filtered after cooling to room temperature. The entire filtrate was then concentrated by rotary evaporation. After removing the protein using the Sevag method, CCP was obtained after ethanol precipitation and lyophilization.

### 2.3. Structural Characterization of CCP

#### 2.3.1. The Content of Polysaccharides and Uronic Acid

The polysaccharide content of CCP was quantified using the phenol–sulfuric acid method. The uronic acid content was estimated using the carbazole–sulfuric acid method, with glucuronic acid as the standard.

#### 2.3.2. UV-Vis Spectroscopy Analysis

CCP (1 mg) was dissolved in ultrapure water (2 mL) to obtain a 0.5 mg/mL polysaccharide solution, which was analyzed using a UV–visible spectrophotometer (756PC, Sunny Hengping Instrument, Shanghai, China) within the wavelength range of 200–400 nm. Adenosine triphosphate (ATP) and bovine serum albumin (BSA) were used as positive controls with peaks at 260 nm and 280 nm.

#### 2.3.3. Fourier-Transform Infrared (FT-IR) Analysis

CCP was determined by FT-IR with a Fourier transform infrared spectrometer (NicoletiS5, Thermo Fisher Scientific, MA, USA). Briefly, 2 mg of CCP was mixed with 200 mg of KBr and pressed into pellets, which were performed within the wave ranges of 4000–500 cm^−1^.

#### 2.3.4. Molecular Weight Determination

The molecular weight distribution of CCP was measured by high-performance gel permeation chromatography (HPGPC). CCP was dissolved in 0.2 M NaCl solution and loaded into an HPLC system (LC-10A, Shimadzu, Kyoto, Japan) equipped with two BRT105-103-101 gel columns (8 mm × 300 mm). The mobile phase was 0.2 M NaCl run at a flow rate of 0.8 mL/min, and the column temperature was maintained at 40 °C.

#### 2.3.5. Monosaccharides Composition Analysis

CCP (5 mg) was hydrolyzed with trifluoroacetic acid (3 M) at 120 °C for 3 h to be converted into acetylated derivatives, which were further determined by ion chromatography (ICS5000, Thermo Fisher, Waltham, MA, USA) equipped with a Dionex CarbopacTMPA20 (3 × 150 mm). The column temperature was set at 30 °C, and the mobile phases of A (H_2_O), B (15 mM NaOH), and C (15 mM NaOH and 100 mM NaOAc) with a flow rate of 3 mL/min were used.

#### 2.3.6. Scanning Electron Microscope (SEM) Analysis

SEM equipment (JSM-7610Fplus, JEOL, Tokyo, Japan) was used to observe the subtle morphological structures and properties of the CCP. The samples were glued onto a specimen stub with a thin layer of platinum coating under high-vacuum conditions at an acceleration voltage of 15 kV.

#### 2.3.7. Nuclear Magnetic Resonance (NMR) Analysis

The NMR spectra were analyzed using Bruker Avance NEO 400 and Quantum-I plus 600 (Bruker, Rheinstetten, Germany) operating at 400 MHz (^1^H) and 600 MHz (^13^C), respectively. CCP powder (50 mg) was dissolved in deuterium oxide (D_2_O, 99%), and the NMR spectra were acquired at room temperature using deuterated acetone as the internal standard. The acquisition parameters were carefully optimized to ensure high spectral resolution and accurate chemical shift assignments.

### 2.4. In Vitro Assay

#### 2.4.1. Cell Culture

HUVECs and RAW264.7 cells were obtained from the Traditional Chinese Medicine Research Institute (Guangdong Pharmaceutical University) and cultured in DMEM containing 1% penicillin–streptomycin and 10% FBS. All cells were cultured at 37 °C with 5% CO_2_ in an incubator.

#### 2.4.2. Cell Survival Analysis

To determine the optimal glucose concentration for establishing an HG damage model of HUVECs, cells (1 × 10^4^ cells/well) were treated with three concentrations of D-glucose (11 mM, 22 mM, and 33 mM) for 24 h, and cell survival viability was assessed using CCK8 assays. The survival rate of the normal glucose group (NG, containing 5.5 mM D-glucose) was used as a control, and the survival rates of cells in the other groups were compared with that of the normal group.

To evaluate CCP cell proliferation against HG injury, HUVECs (1 × 10^4^ cells/well) were treated with different concentrations of CCP (25, 50, 100, or 200 μg/mL) for 24 h at HG levels (33 mM), and CCK8 was used to measure cell viability.

#### 2.4.3. Cell Scratch Assay

Scratch wound migration assay was performed to detect HUVEC migration. Cells were seeded into 6-well plates and cultured for 12 h. After the cells reached 80% density, the medium was exchanged for serum-free medium for another 24 h. Subsequently, a straight scratch was made in each replicate well of the monolayer cells using sterile pipette tips, and the cells were treated with NG (containing 5.5 mM D-glucose), HG (containing 33 mM D-glucose), CCP plus HG (containing 25, 50, 100, and 200 μg/mL CCP plus 33 mM D-glucose). The scratch-healing areas were photographed using a CKX41 cell inverted microscope (Olympus, Tokyo, Japan) at 0 and 24 h. The migration rate was calculated using Equation (1):

(1) $$\mathrm{Migration}\;\mathrm{area}\left(\%\right)=\left(A_{0}-A_{24}\right)/A_{0}\times 100\%$$

#### 2.4.4. Tube Formation Assay

The angiogenic capacity of HUVECs was assessed using a tube formation assay. HUVEC suspensions (4 × 10^4^ cells/well) were treated under different conditions for 24 h, then 100 μL of cell suspension was added onto the gelled matrix gel. After 6 h of cultivation, images were captured under an optical microscope and analyzed using ImageJ software (1.54g).

#### 2.4.5. Anti-Inflammatory Activity Assay

qPCR was performed to measure the mRNA expression levels of *IL-6*, *IL-1β*, *TNF-α*, *Arg-1*, *IL-10*, and *TGF-β* in macrophages. RAW 264.7 cells were first seeded into 12-well plates and cultured until the cell density reached 80%. CCP (100, 200, and 400 μg/mL) was simultaneously added with LPS (1 μg/mL) to RAW264.7 cells and co-cultured for 3 h, and total cellular RNA was extracted for qPCR analysis of *IL-6*, *IL-1β*, *TNF-α*, *Arg-1*, *IL-10*, and *TGF-β*. The primer sequences employed in this study are listed in Table 1.

Enzyme-linked immunosorbent assay (ELISA) was performed to measure the secreted IL-1β, TNF-α, Arg-1, and IL-10 in macrophages. Raw264.7 cells were seeded in 48-well plates at 1 × 10^5^ cells/well. After incubating the cells with LPS alone or LPS plus CCP at the indicated concentrations, the supernatant was collected and analyzed according to the manufacturer’s instructions.

Western blotting was used to detect the expression and phosphorylation levels of STAT3. Total protein was extracted from RAW264.7 cells after treatment, and the protein concentration was determined using a BCA kit. Western blotting was performed according to standard procedures. The image of blotted proteins was obtained using a BIO-RAD imaging system (Bio-Rad, Hercules, CA, USA) and further quantified using Image J.

### 2.5. Antibiofilm Experiment

*S. aureus* and *E. coli* were added to a 96-well plate and incubated at 37 °C for 48 h to form biofilms. After washing with PBS, different concentrations of CCP solution were added, and the plate was further incubated at 37 °C. The biofilms were fixed with methanol for 15 min after washing with PBS. Then, 100 µL of 0.1 % crystal violet solution was added and incubated for 30 min. After removing excess dye, images were captured. For quantitative analysis, 33% acetic acid was added to each well to dissolve the remaining dye, and a microplate reader was used to measure the absorbance of the dissolved dye at a wavelength of 595 nm to quantify biofilm biomass.

### 2.6. Antibacterial Experiment

*S. aureus* and *E. coli* were cultured overnight in LB medium at 37 °C with 180 rpm shaking. Bacterial suspensions in the logarithmic phase of growth were collected and incubated with various concentrations of CCP (0, 100, 200, and 400 μg/mL) at 37 °C for 30 min. For hydrogels, 0.1 g of different types of hydrogels were incubated in 1 mL of LB medium for 24 h, followed by co-culture with bacterial suspensions for 30 min. Finally, 100 μL of mixed suspensions from each treatment group was streaked onto LB agar plates and incubated at 37 °C for 24 h, and bacterial colony counts were determined to evaluate the antimicrobial activity of CCP.

### 2.7. Preparation and Characterization of Hydrogels

#### 2.7.1. Preparation of Hydrogels

A mixture of 2% (*w*/*v*) carbomer (CBM) 940 and 2.5% (*w*/*v*) sodium carboxymethylcellulose (CMC-Na) was prepared at a strict volume ratio of 1:1. Subsequently, these mixtures were blended with different concentrations of CCP solution and stirred continuously for 30 min. The pH of the resulting mixture was adjusted to 7.0 using triethanolamine. As shown in Table 2, the CBM/CMC-Na composite hydrogels were formulated into three groups, differing in the presence or absence of CCP.

#### 2.7.2. Characterization of Hydrogels

The prepared hydrogels were freeze-dried, and the structure and chemical linkages of the hydrogels were determined by FT-IR according to the method described above. For morphological analysis, the freeze-dried samples were sectioned and mounted onto a substrate, then gold-sputtered for SEM imaging.

#### 2.7.3. Water Retention

The obtained hydrogels were individually placed into 2 mL centrifuge tubes and incubated at 37 °C, and the initial weight of each hydrogel sample was recorded as *M*_0_. At each predetermined time point, the weight of each hydrogel was recorded as *M*_1_. The water retention (W_R_) of hydrogel was calculated using Equation (2):

(2) $$W_{R}=1-(M_{0}-M_{1})/M_{0}\times 100\%$$

#### 2.7.4. Swelling Rate

Hydrogel swelling behavior was evaluated using the weighing method. Briefly, the initial weight of each hydrogel was recorded as *W*_0_. After swelling at room temperature in distilled water, excess water from the remaining surface was wiped with tissue paper, and the mass of the swollen hydrogels was measured as *W*_1_ at predetermined time intervals. The hydrogel swelling rate was calculated using Equation (3):

(3) $$\mathrm{Swelling}\;\mathrm{rate}=(W_{1}-W_{0})/W_{0}\times 100\%$$

#### 2.7.5. Release of CCP from Hydrogel

0.5 g of CCP_0.11_/CCHG or CCP_0.55_/CCHG was transferred into 1.5 mL phosphate-buffered saline (pH 7.4) in a 2 mL centrifuge tube and placed in a constant-temperature shaker with agitation parameters set at 37 °C and 100 rpm, respectively. Then, 1 mL of the solution was removed at predetermined time points (1, 2, 4, 6, 8, 10, 12, and 24 h) for analysis, followed by replenishing 1 mL of fresh PBS solution until the concentrations of CCP plateaued. The CCP contents in the collected supernatants were quantified via the phenol–sulfuric acid method: the absorbance of each sample solution was measured at 490 nm using a microplate reader, and the CCP concentration was derived from a pre-calibrated CCP standard curve. Cumulative drug release was calculated using Equation (4):

(4) $$Q_{n}=\frac{C_{i}\times V+\sum_{i=1}^{n-1}\left(C_{i}\times v\right)}{M}\times 100\%$$

*Q_n_*: Cumulative release degree at the indicated time point. *Cn*: Drug concentration measured in the sample taken at the indicated time point. *V*: Initial solvent volume. *C_i_*: Drug concentration was measured at the indicated time point. *v*: Volume of each sample. *M*: Total drug mass in the hydrogel.

#### 2.7.6. Rheological Analysis

Hydrogel rheological properties were characterized using a modular compact rheometer (Anton Paar GmbH, Graz, Austria). The hydrogel sample was placed between 25 mm parallel plates, and the gap adjusted to 1 mm; the rotational speed was set at 6.3 rad/s, and all measurements were performed at 25 °C. Strain sweep tests were conducted over a strain range of 0.1–100%, oscillation time sweep tests were carried out under low strain (0.5%) and high strain (100%) conditions, and time sweep and frequency sweep measurements were performed with a fixed strain of 0.5%, respectively. Additionally, viscosity was determined at shear rates ranging from 0.01 to 1000 s^−1^.

### 2.8. Biocompatibility Evaluation

Twenty male SPF-grade Sprague Dawley (SD) rats (4–5 weeks old) were purchased from Zhuhai BesTest Bio-Tech Co., Ltd. (Zhuhai, China), and the animal experiments were ethically approved by the Animal Experimentation Committee of Guangdong Pharmaceutical University (approval no. SPF2022377).

#### 2.8.1. Skin Irritation Experiment

To ensure the safety of the dosage employed in this study, in vivo skin safety evaluations were performed on rats through visual observation and hematoxylin and eosin (H&E) staining. After removing the hair on the back of the rats, CCHG, CCP_0.11_/CCHG, and CCP_0.55_/CCHG were applied to the back skin of rats, respectively. Visual observations of the skin were conducted, and photographs were captured at 24, 48, and 72 h post-application. Finally, the treated skin tissues were excised for H&E staining to evaluate local tissue damage and inflammatory responses.

#### 2.8.2. Blood Compatibility Test

The hemocompatibility of the hydrogels was evaluated using a hemolysis assay. An amount of 1 g of each hydrogel was individually mixed with 10 mL of 0.9% NaCl solution and incubated at 37 °C for 24 h to prepare hydrogel extracts. Fresh whole blood was centrifuged to isolate red blood cells (RBCs), which were further diluted to 10% with saline and incubated with different hydrogel extracts at 37 °C for 30 min. Finally, the mixtures were centrifuged at 3500 rpm for 5 min, and the absorbance of the resulting supernatant was measured at 542 nm using a microplate reader. The hemolysis rate was calculated using Equation (5):

(5) $$\mathrm{Hemolysis}\;\mathrm{rate}\;(\%)=\frac{{OD}_{sample}-{OD}_{0.9\%\;NaCl}}{{OD}_{Triton\;X-100}-{OD}_{0.9\%\;NaCl}}\times 100\%$$

*OD_sample_*: Absorbance value dissolved in hydrogel supernatant; *OD*_0.9% *NaCl*_: Absorbance value dissolved in physiological saline; *OD*_Triton X-100_: Absorbance value of complete hemolysis.

#### 2.8.3. Cell Compatibility of Hydrogels

HUVECs were treated with gel extracts prepared from cell culture medium containing blank hydrogels or hydrogels containing CCP. CCK-8 was used to assess cell viability or proliferation after 24 h of culture.

### 2.9. The Effect of CCP on Wound Healing in Diabetic Rats

#### 2.9.1. In Vivo Wound Healing Assay

The establishment of a diabetic rat model was performed according to methods described in the literature [27]. After adaptive feeding for 7 days, SD rats were administered a single intraperitoneal injection of 65 mg/kg STZ, and their fasting blood glucose levels exceeding 11.1 mmol/L after 3 and 7 days post-injection were selected for subsequent DW modeling. After anesthesia, two 10 mm full-thickness skin wounds were created in the dorsal region of each rat. Diabetic rats were further randomly divided into four groups: the model group (CCHG), positive group (JWH), low-dose CCP hydrogel group (CCP_0.11_/CCHG), and high-dose CCP hydrogel group (CCP_0.55_/CCHG). All preparations were administered topically to the wounds.

#### 2.9.2. Physiological Indexes and Wound Healing Assay

During the treatment period, fasting blood glucose levels and body weight were measured on days 0, 6, and 12. Wound images were collected and analyzed using ImageJ on days 0, 1, 3, 5, 7, 9, and 11. The wound area and wound healing rate were calculated by Equations (6) and (7), respectively:

(6) $$\mathrm{Wound}\;\mathrm{area}\;\left(\%\right)=\;\frac{\mathrm{Wound}\;\mathrm{area}}{\mathrm{Original}\;\mathrm{wound}\;\mathrm{area}}\times 100\%$$

(7) $$\mathrm{Wound}\;\mathrm{healing}\;\mathrm{rate}\;\left(\%\right)=\frac{\mathrm{Original}\;\mathrm{wound}\;\mathrm{area}-\mathrm{Wound}\;\mathrm{area}}{\mathrm{Original}\;\mathrm{wound}\;\mathrm{area}}\times 100\%$$

#### 2.9.3. H&E and Masson Staining

Rats were sacrificed for histological analysis after treatment. The wounds and their surrounding skin tissues were excised and embedded in paraffin after fixation with 4% paraformaldehyde. The samples were then sectioned and stained with hematoxylin and eosin (H&E) and Masson’s trichrome to observe the inflammatory response, reepithelialization, and collagen formation. The main organs of rats after treatment, including the heart, liver, spleen, lung, and kidney, were removed for H&E staining.

#### 2.9.4. Immunohistochemistry Staining

Tissue sections were prepared by antigen retrieval and endogenous peroxidase inactivation. The sections were incubated overnight at 4 °C with primary antibodies against vWF, TGF-β1, CD31, VEGF, TNF-α, IL-6, and IL-10. Following PBS washing, the sections were incubated with horseradish peroxidase-conjugated secondary antibodies at room temperature for 50 min, followed by 3,3′-diaminobenzidine and hematoxylin staining. IHC signals were analyzed using ImageJ software.

### 2.10. Statistical Analysis

All data are presented as the mean ± SEM. Data were processed and analyzed using GraphPad 9.0. Statistical analysis was performed using one-way analysis of variance (ANOVA) or *t*-tests. Differences were considered statistically significant at *p*-values less than 0.05, * *p* < 0.05, ** *p* < 0.01, *** *p* < 0.001.

## 3. Results and Discussion

### 3.1. Extraction and Characterization of CCP

Numerous studies have found that the biological activity of polysaccharides is closely related to their extraction process, purity, molecular weight, monosaccharide composition, and configuration [30]. CCP was successfully isolated as a yellowish powder via hot water extraction, ethanol precipitation, protein removal, dialysis, and lyophilization, with a yield of ~4.3%.

**Figure 1:**
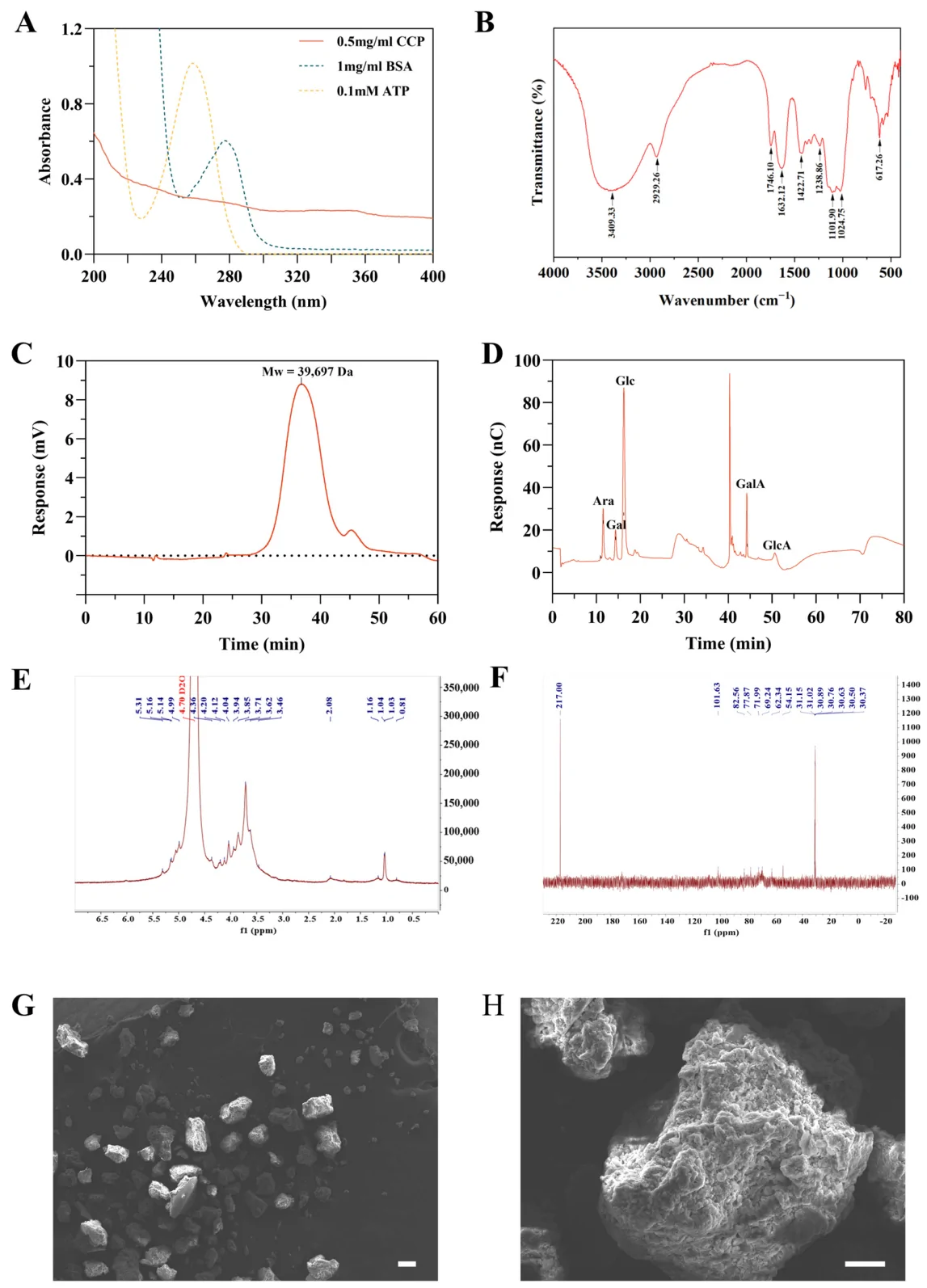
Physicochemical properties of CCP. (**A**) UV-Vis spectra of CCP. (**B**) FT-IR spectra of CCP. (**C**) HPLC chromatogram of CCP acid hydrolysate derivatives. (**D**) Elution profile of CCP on HPGPC. (**E**) ^1^H NMR (400 MHz, in D_2_O) and (**F**) ^13^C NMR (600 MHz, in D_2_O) spectra of CCP. SEM image of CCP (**G**) at low magnification (scale bar = 100 μm) and (**H**) local high-magnification image (scale bar = 50 μm).

Subsequently, the phenol–sulfuric acid and sulfuric acid-carbazole methods were employed to determine the glucose and uronic acid contents of CCP, respectively. The glucose and uronic acid contents of CCP were calculated as 67.6% and 53.8% according to the standard curves drawn from various concentrations of standards (Figure S1). As the main active ingredient of polysaccharides, a higher content of uronic acid might suggest more potent bioactivities, such as antioxidant and immunomodulatory effects [31]. In comparison with other polysaccharides, CCP was enriched with uronic acid, which endowed it with more biological properties. Meanwhile, no ATP and BSA characteristic absorption peaks were observed on the UV-vis spectra of CCP at a concentration of 0.5 mg/mL, indicating that CCP contained no protein or nucleic acid and had high purity (Figure 1A).

The conformation and main functional groups of CCP were identified using FTIR spectroscopy (Figure 1B). As indicated in the spectrum, a prominent broad absorption peak was detected at 3409.33 cm^−1^, which can be assigned to the stretching vibrations of hydroxyl (-OH) groups [32]. Meanwhile, a weak absorption peak was observed at 2929.26 cm^−1^, corresponding to the stretching vibrations of C-H bonds [33]. The coexistence of these two peaks was mainly due to the presence of polysaccharide structures [34]. Additionally, the peaks around 1746.10 cm^−1^ and 1632.12 cm^−1^ belonged to the stretch band of C=O, which corresponded to esterification and free carboxyl of CCP [35]. The characteristic stretching vibration peak of C-H appeared at 1422.71 cm^−1^. The presence of these C=O- and C-H-related absorption bands collectively suggests the existence of glucuronic acid residues in the CCP, which is consistent with the structural characteristics of many natural polysaccharides that possess uronic acid moieties and exhibit specific biological activities [36].

Moreover, the elution peak of CCP from HPGPC was single, steep, and symmetric, reflecting the trait of homogeneous distribution, and further confirming CCP as a homogeneous polysaccharide (Figure 1C). Using a standard curve generated from dextran standards of different molecular weights (Figure S2), the average molecular weight of CCP was determined to be 39,697 Da with a corresponding peak area of nearly 100.00% (Table 3). The CCP monosaccharide composition was further determined based on the ion chromatogram of 16 mixed monosaccharide standards (Figure S3), and the results reveal that CCP was mainly composed of glucose, galacturonic acid, arabinose, and galactose, with molar percentages of 47.40%, 21.30%, 14.20%, and 10.80%, respectively (Figure 1D and Table 4).

Meanwhile, NMR spectroscopy was used to further elucidate the monosaccharide composition and structural details of CCP. The ^1^H NMR spectrum showed that the chemical shifts of CCP were mainly distributed between 3.0 and 5.5 ppm, consistent with the typical range of polysaccharides (Figure 1E) [37]. Specifically, four prominent signals were observed at 4.99, 5.14, 5.16, and 5.31 ppm, which were attributed to arabinose, galactose, mannose, and glucose, respectively [38,39]. In addition, three anomeric proton signals appeared at 5.0–5.5 ppm, suggesting the existence of α-configurations. A strong signal at 4.70 ppm reflects residual solvent. The ^13^C NMR spectrum demonstrated distinct anomeric signals at 101.63 ppm, characteristic of α-configuration and associated with O-glycosidic linkages (Figure 1F) [40]. Signals at 69.24, 71.99, 77.87, and 82.56 ppm corresponded to C2–C5 of the glycosyl ring, while the signal at 62.34 ppm confirmed the presence of C6 in galactose within the CCP polysaccharide chain [41]. Collectively, these results demonstrate the presence of various distinct glycosidic residues in CCP. Many studies have shown that the biological activity of natural polysaccharides is closely related to their monosaccharide compositions [42]. Previous studies have demonstrated that arabinose-containing polysaccharides was found to be able to enhance immune activity, and the potential antioxidant activity of *Radix Sophorae tonkinensis* polysaccharides was mainly attributed to their high galactose content. These studies perhaps implied that CCP had the ability to promote wound healing in diabetic patients. Finally, SEM observation revealed that CCP particles exhibit irregularly arranged bulk structures with compact morphologies and rough surface (Figure 1G,H).

### 3.2. CCP Promotes Proliferation, Migration, and Microtubule Formation in HUVECs

**Figure 2:**
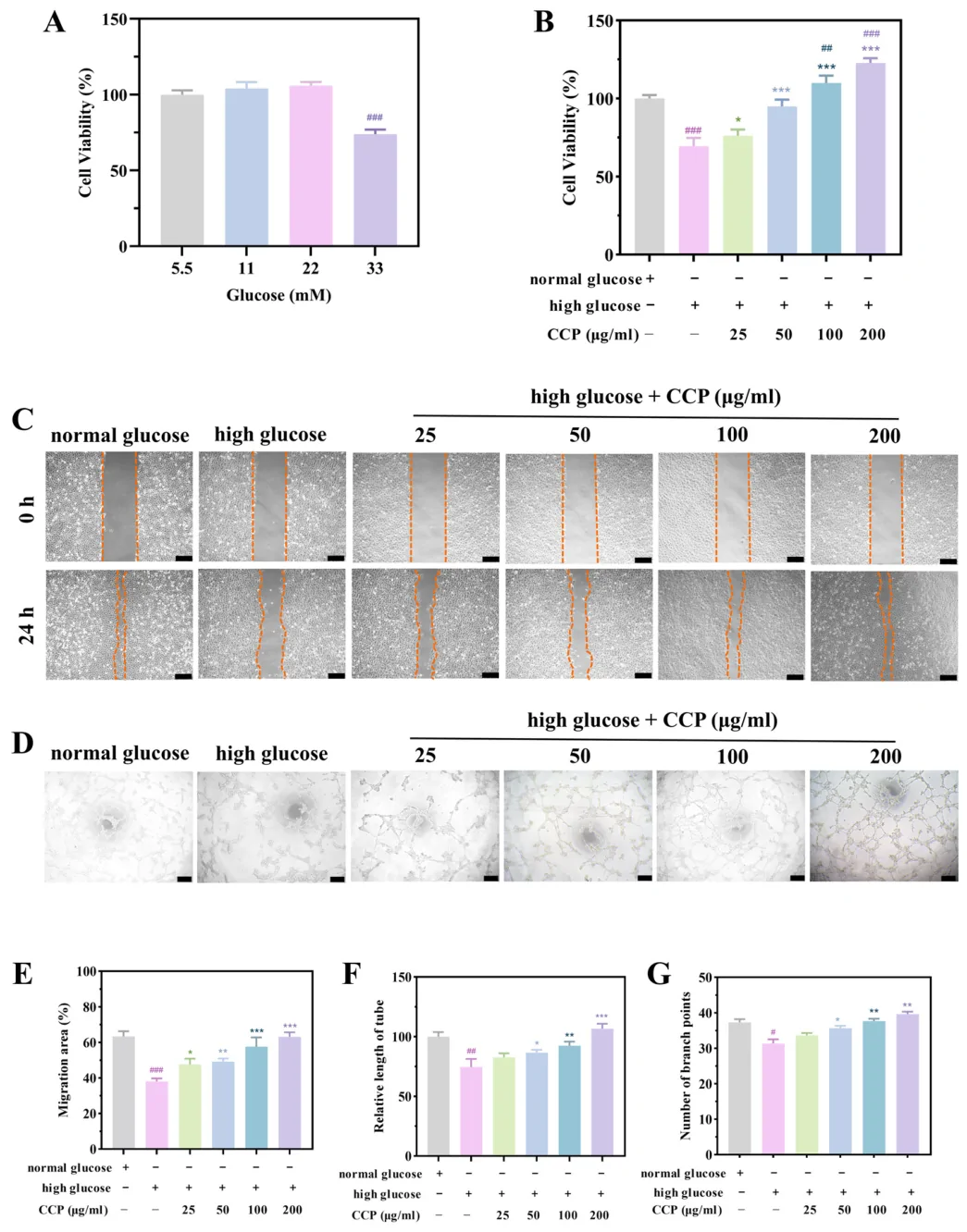
CCP improved HUVEC proliferation, migration, and tube formation in a high-glucose environment. (**A**) Cell viability of HUVECs after treatment with various concentrations of glucose for 24 h. (**B**) CCP reverses high-glucose-induced inhibition of HUVECs proliferation. (**C**) The migration ability of CCP on HUVECs in a high-glucose environment was detected by scratch assay (scale bar = 100 μm). (**D**) The angiogenic abilities of the indicated treated HUVECs measured by tube formation assays (scale bar = 200 μm). (**E**) Quantitative analysis of HUVECs migration rate. Quantification analysis of (**F**), the total segment length, and (**G**), the number of nodes observed in HUVECs. Data are expressed as standard error of the mean (*n* = 3). ^#^ *p* < 0.05, ^##^ *p* < 0.01, ^###^ *p* < 0.001 versus the normal glucose group. * *p* < 0.05, ** *p* < 0.01, *** *p* < 0.001 versus the high glucose group.

Persistent hyperglycemia is already considered a predominant characteristic of DW, which perpetually impairs vascular endothelial cell migration, tube formation, and hinders neovascularization, a pivotal process for effective wound healing [43]. HUVECs are widely used to study vascular endothelial function and angiogenesis mechanisms, specifically in the context of diabetic vasculopathy [44]. To determine the optimum concentration for generating the HG injury cell model in vitro, HUVECs were treated with various glucose concentrations, and their cell viability was tested using the CCK8 assay. As shown in Figure 2A, HUVECs exposed to hyperglycemia (33 mmol/L) resulted in a significant decrease in cell viability to approximately 26% compared with the normoglycemic control. Thus, this glucose concentration was selected to establish the HG-induced cell impairment model for subsequent studies. Next, to evaluate the protective effect of CCP on HG-induced cell injury, HUVECs maintained in HG were treated with CCP at varying concentrations, and the results indicate that CCP dose-dependently alleviated cell damage induced by HG (Figure 2B). Remarkably, the 200 μg/mL CCP treatment group exhibited impressive rescue activity and showed better cell viability compared with the normal group.

Next, the scratch wound healing assay and tube formation on Matrigel were employed to evaluate the effect of CCP on the migration and tube formation capacities of HG-injured HUVECs. As shown in Figure 2C, the cell migration rate in the HG model group was significantly lower than that in the normal control group at 24 h post-scratch, whereas CCP treatment dose-dependently increased the migration rate of the model group. Quantitative analysis results show that the migration ability of the 200 μg/mL CCP-treated group was significantly increased and recovered compared with that of the normal control group (Figure 2E). Consistent with previously reported results, we observed that HG significantly inhibited endothelial cell tube formation and HUVECs only formed sparse and discontinuous tube-like structures [45]. As expected, CCP treatment remarkably improved the tube formation capacity of HG-injured HUVECs, resulting in the formation of more intact and dense vascular network structures (Figure 2D). Quantitative analysis further confirmed that CCP significantly increased the total tube length and the number of branch nodes in a dose-dependent manner (Figure 2F,G). Our results systematically demonstrate that CCP could alleviate HG-induced HUVEC injury and promote their proliferation, migration, and tube formation abilities in a dose-dependent manner, suggesting that CCP has potential as a natural active ingredient for promoting neovascularization and accelerating DW healing.

### 3.3. CCP Regulates Inflammatory Cytokines

**Figure 3:**
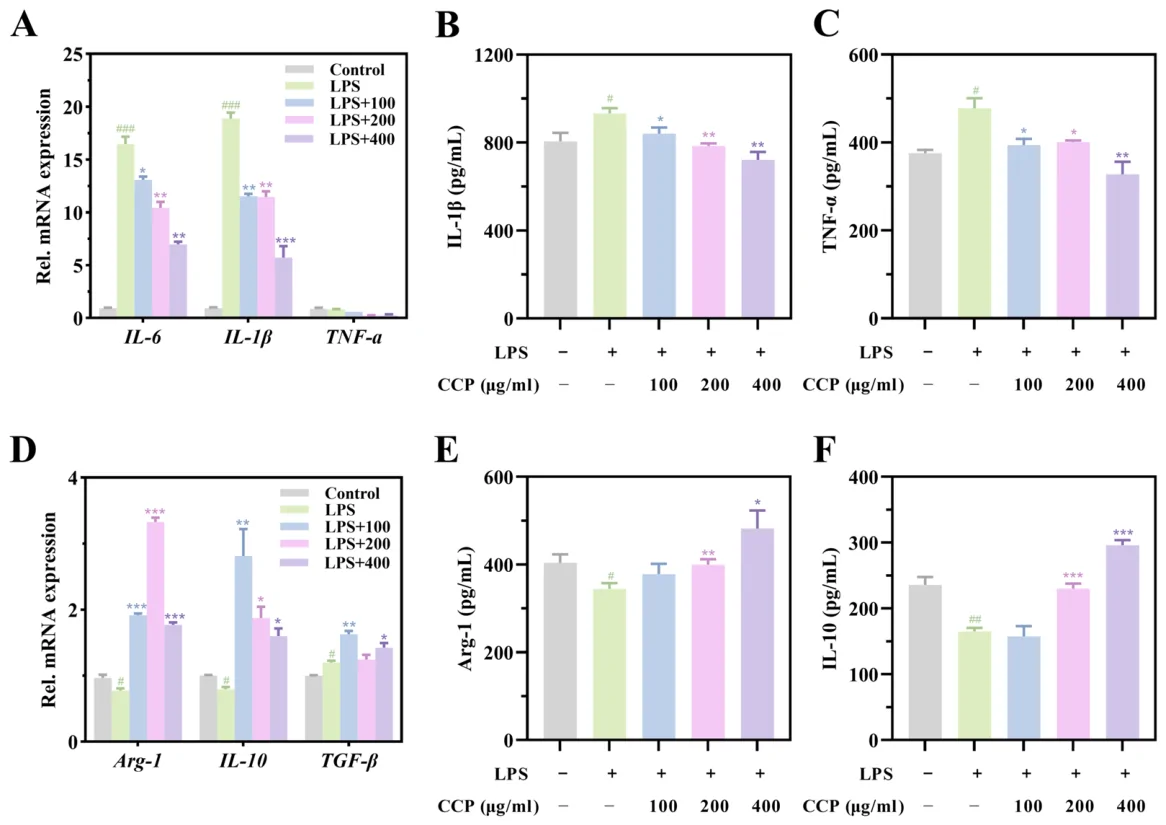
Anti-inflammatory properties of CCP in vitro. (**A**) CCP inhibits the expression of M1 macrophage markers (*IL-6*, *IL-1β*, and *TNF-α*). ELISA detection of M1-associated cytokines (**B**) IL-1β and (**C**) TNF-α secretion from RAW264.7 after indicated treatments. (**D**) Effects of CCP on the expression of M2 macrophage markers (*Arg-1*, *IL-10*, and *TGF-β*). ELISA detection of M2-associated cytokines (**E**) Arg-1 and (**F**) IL-10 secretion from RAW264.7 after indicated treatments. Data are expressed as standard error of the mean (*n* = 3). ^#^ *p* < 0.05, ^##^ *p* < 0.01, ^###^ *p* < 0.001 versus the control group. * *p* < 0.05, ** *p* < 0.01, *** *p* < 0.001 versus the LPS group.

During normal wound healing, pro-inflammatory M1 macrophages predominate early on, whereas anti-inflammatory M2 macrophages promote inflammation resolution and tissue regeneration in later phases [46]. The macrophages in the DM healing microenvironment tend to maintain the M1 phenotype, which leads to persistent and excessive inflammation [47]. Modulating macrophage polarization to reprogram the inflammatory milieu of DW is therefore critically important for promoting wound repair, as it targets the core pathogenic factors underlying impaired healing in these wounds [48]. To further explore the potential role of CCP in promoting M1 to M2 macrophage polarization, we detected the expression levels of pro-inflammatory factors (*IL-6*, *IL-1β*, *TNF-α*) and anti-inflammatory cytokines (*Arg-1*, *IL-10*, *TGF-β*) in LPS-stimulated RAW 264.7 cells. As shown in Figure 3A, LPS induced upregulation of *IL-6* and *IL-1β*, which promoted M1 macrophage polarization and inflammation. Notably, CCP dose-dependently reduced the expression of *IL-6* and *IL-1β* in LPS-simulated RAW cells and also downregulated the mRNA levels of *TNF-α*. ELISA detection of cell supernatants further confirmed that CCP suppressed the LPS-induced secretion of IL-1β and TNF-α in a dose-dependent manner (Figure 3B,C). In contrast, CCP treatment significantly upregulated the expression of *Arg-1*, *IL-10* and *TGF-β* (Figure 3D). Consistent with this, extracellular secretion of Arg-1 and IL-10 derived from macrophages was also substantially elevated after CCP treatment (Figure 3E,F). These results demonstrate CCP efficiently promotes the polarization of the LPS-induced macrophage line from a pro-inflammatory M1 phenotype to an anti-inflammatory M2 phenotype, while suppressing the secretion of pro-inflammatory cytokines and elevating the production of anti-inflammatory cytokines. Notably, accumulating evidence suggested the regulatory effects of polysaccharides on macrophages [49]. Lentinan has been shown to promote the polarization of M1-type macrophages toward the M2 phenotype [50]. Astragalus polysaccharide was found to be capable of downregulating M1-related genes, including *NF-κB*, and thus promoting macrophage M2 polarization [51]. These findings were consistent with our findings, indicating that the regulation of macrophage polarization may represent a common anti-inflammatory mechanism shared by natural polysaccharides. Given the central role of STAT3 in regulating macrophage polarization, we further explored the regulatory effect of CCP on STAT3 expression. As shown in Figure S4, LPS stimulation significantly elevated the phosphorylation level of STAT3, whereas CCP treatment dose-dependently reversed LPS-induced excessive STAT3 phosphorylation. In the DW microenvironment, abnormal STAT3 activation drives macrophage polarization toward the M1 phenotype, triggers the overexpression of pro-inflammatory cytokines, and consequently maintains persistent local inflammation and inhibits wound repair [52]. Our results suggest that CCP may promote the transformation of macrophages from the M1 to the M2 phenotype by inhibiting STAT3 overactivation, thereby reducing inflammatory infiltration and reshaping the microenvironment conducive to DW healing. Collectively, our results demonstrate that CCP exerted dual regulatory effects on macrophage polarization by inhibiting M1 and promoting M2 polarization, which alleviated persistent inflammation in DW and thereby allowed the establishment of a favorable microenvironment for wound repair.

### 3.4. Antibiofilm and Antibacterial Effect of CCP

**Figure 4:**
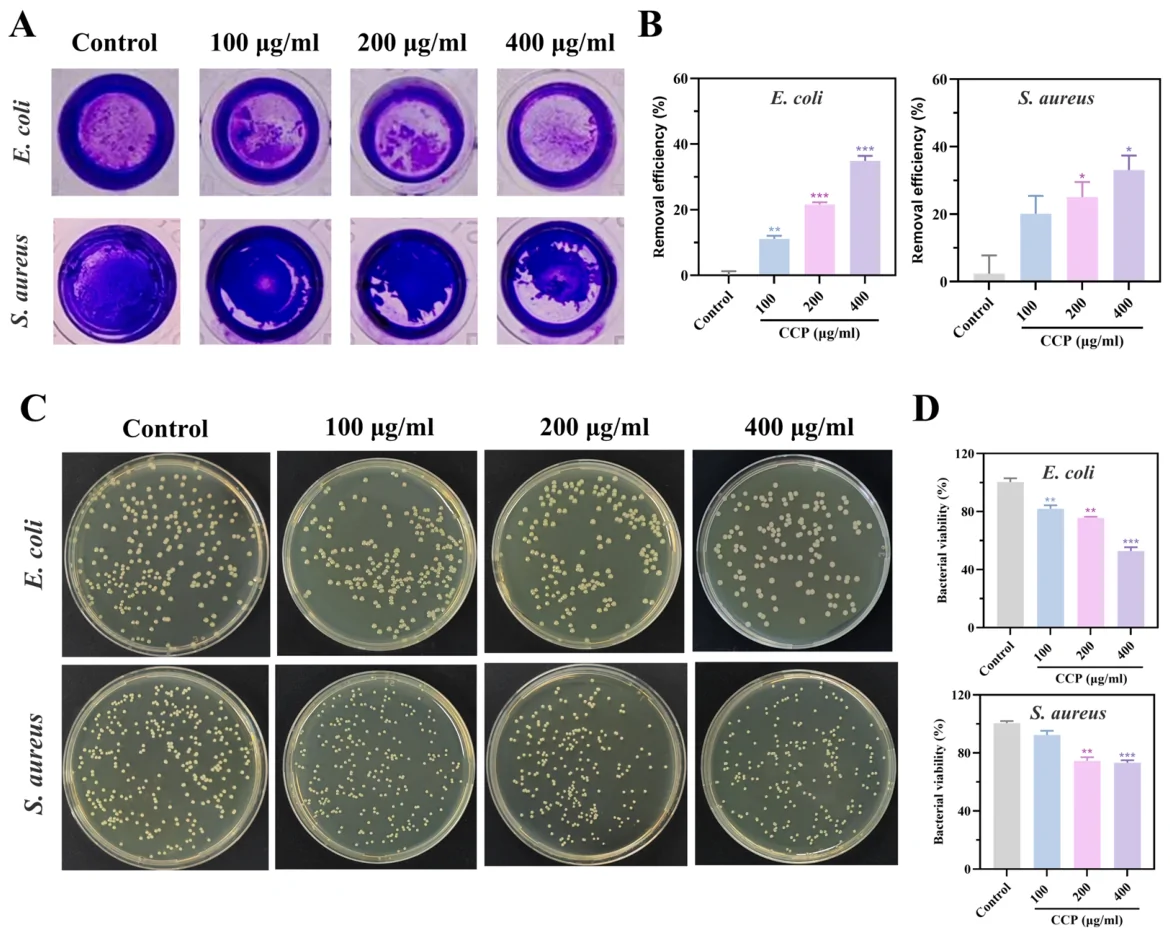
Antibacterial activity of CCP. (**A**) Photographs of crystal violet-stained biofilms of *E. coli* and *S. aureus* after different treatments. (**B**) Biofilm clearance induced by CCP was quantified by determining biofilm biomass at OD595 after crystal violet staining. (**C**) Photographs of plate count agar from different groups. (**D**) Corresponding antibacterial rates against *E. coli* and *S. aureus*. Data are expressed as standard error of the mean (*n* = 3). * *p* < 0.05, ** *p* < 0.01, *** *p* < 0.001 versus the control group.

The hyperglycemic microenvironment of DW offers a permissive environment for bacterial colonization, invasion, and overgrowth, triggering biofilm formation [53]. The formed biofilm physically impedes the penetration of topical antimicrobials into wound tissues, which perpetuates persistent local inflammation, inhibits tissue regeneration and re-epithelialization, and eventually results in intractable and recurrent DW infections. Accordingly, antibiofilm and antibacterial properties have become critical considerations for the design and development of advanced wound dressings [54]. Antibiotics are commonly used for infected DW, but their abuse and resistance have become a serious problem worldwide [55]. Recently, the search for and development of safe and green antibiotic alternatives from natural herbs have gained attention. The inhibitory effect of CC on microorganisms has been used in traditional medicine for thousands of years [56]. However, it is not known whether CCP has antimicrobial activity. In this study, two types of bacteria (*E. coli* and *S. aureus*), the most common pathogenic bacteria isolated from DW, were used to evaluate the antibacterial properties of CCP. The crystal violet staining assay verified that CCP treatment markedly inhibited biofilm formation by the two bacterial strains in a dose-dependent manner (Figure 4A). At a CCP concentration of 400 μg/mL, the biofilm biomass of *E. coli* and *S. aureus* significantly decreased, with eradication efficiencies of 33.06% and 34.87%, respectively (Figure 4B). These results confirm that CCP has a certain bacterial biofilm elimination capacity. The antibacterial effects of CCP against these two strains were further investigated. For *E. coli*, CCP significantly reduced the number of colonies compared with the control group in a dose-dependent manner, with inhibitory rates of approximately 24.24% and 47.17% at concentrations of 200 μg/mL and 400 μg/mL, respectively (Figure 4C). Similarly, 200 μg/mL CCP exhibited an inhibitory rate of 25.43% against *S. aureus*. However, doubling the CCP concentration only slightly increased the inhibitory effect to 26.73% (Figure 4D). These results indicate that CCP exerts moderate antibacterial activity against both *E. coli* and *S. aureus*, with better antibacterial efficacy against the former than the latter. This phenomenon might be related to the intrinsic structural differences between the two bacterial strains. The anionic groups (e.g., uronic acids) of plant polysaccharides could interact with cationic components in the LPS of *E. coli*, thereby disrupting outer membrane integrity and enabling cytoplasmic penetration [57]. In contrast, *S. aureus* consists of a robust barrier cross-linked by a peptidoglycan network and teichoic acid, which could hinder high-molecular-weight polysaccharides penetration and diminish their inhibitory efficacy [58]. The inhibitory activity of CCP against these two strains suggests it may effectively alleviate the bacterial burden in DW, thereby creating a microenvironment conducive to DW repair.

### 3.5. Fabrication and Physicochemical Characterization of CCP Hydrogels

**Figure 5:**
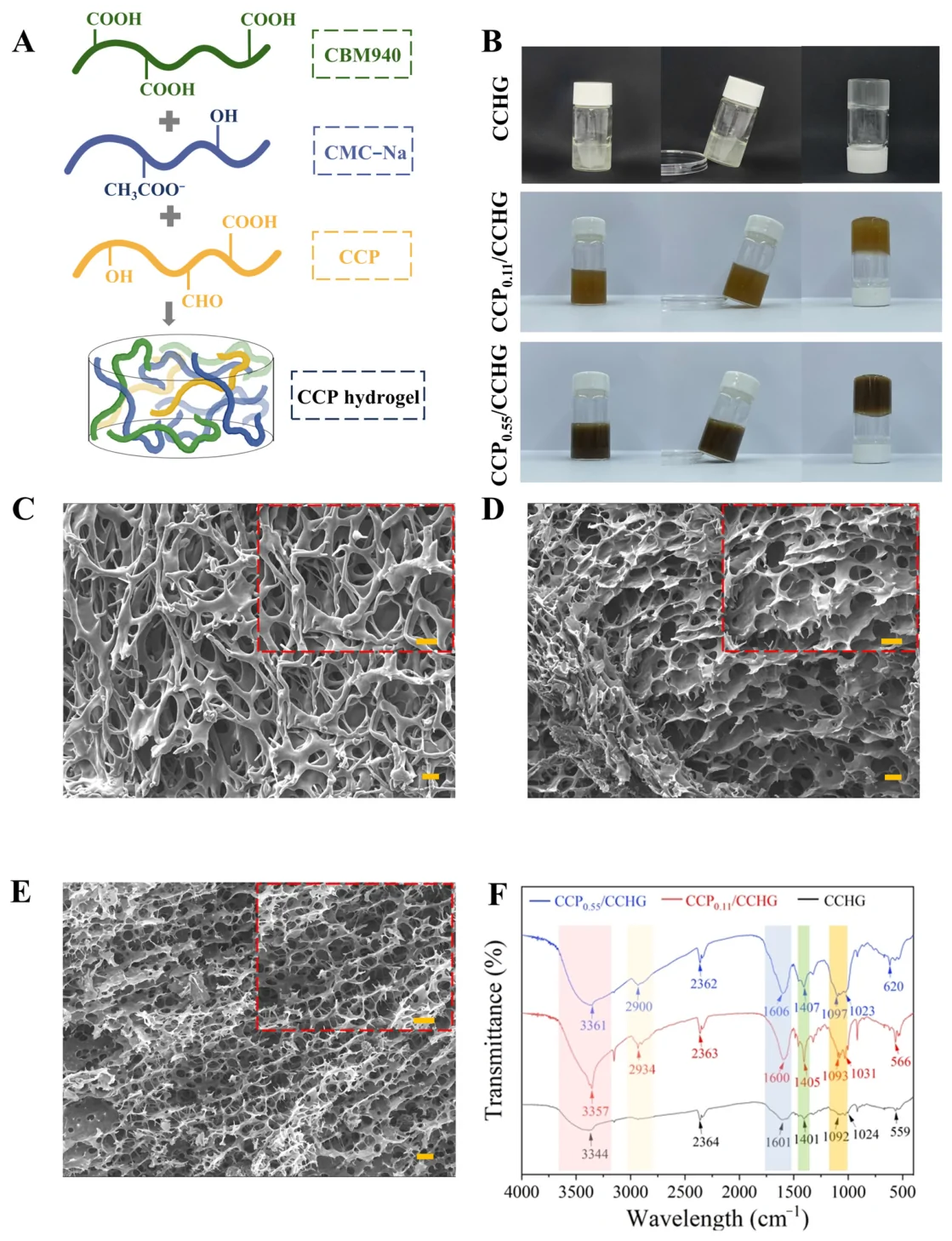
Morphology and structure characterizations of hydrogels. (**A**) Schematic diagram of the fabrication process of the CCP/CCHG hydrogel. (**B**) The gelling processes of CCHG, CCP_0.11_/CCHG and CCP_0.55_/CCHG hydrogels were determined using an inverting experiment. SEM images of (**C**) CCHG, (**D**) CCP_0.11_/CCHG and (**E**) CCP_0.55_/CCHG. The Insert images in (**C**–**E**) are local magnification images (scale bar = 25 μM). (**F**) FT-IR spectra of the prepared CCHG, CCP_0.11_/CCHG, and CCP_0.55_/CCHG hydrogels.

CBM940 and CMC-Na have been widely employed as hydrogel matrices for drug delivery, primarily attributed to their exceptional biosafety profiles and favorable hydrodynamic properties [59]. The preparation procedure for the CCP hydrogels is presented in Figure 5A. CMC-Na and CBM940 were co-incorporated to fabricate the hydrogel matrix, wherein an in situ hydrogen-bonding network was formed via intermolecular interactions between the hydroxyl and carboxyl groups of the two components. Subsequent CCP incorporation increased hydrogen bond density, attributed to its inherent abundant galacturonic acid moieties. As expected, aqueous solutions of CBM940, CMC-Na, and CCP were capable of rapidly forming a homogeneous and stable hydrogel structure without downward flow (Figure 5B). Additionally, the CCP hydrogel was observed to exhibit a uniform brown color with the hue deepening as the CCP dosage increased, which indicated that AMP was uniformly distributed throughout the whole gel matrix.

Next, the microstructures of all hydrogel types were investigated using SEM. As shown in Figure 5C, the CCHG hydrogel possessed irregular porous network microstructures. In contrast, the incorporation of CCP led to the formation of a more compact and uniform porous architecture, and the pore size of the hydrogels gradually decreased with the increase in CCP content (Figure 5D,E). Notably, observations obtained through high-magnification imaging revealed that the internal structure of the CCP-containing hydrogel groups was a smooth surface, and no deposited particles were observed, confirming the successful embedding of CCP polysaccharides into the pristine hydrogel matrix. Meanwhile, CCP added to the CCHG hydrogel group also transformed the dendritic wall structures into broader planar wall structures. Benefiting from these structural variations, CCP hydrogels have a large specific surface area, which is conducive to the adsorption of bioactive molecules and enhances their interaction with cells and tissues.

FTIR spectroscopy was also used to characterize the chemical structure of the prepared hydrogels (Figure 5F). The CCP-loaded hydrogels showed broad absorption peaks at 3353 cm^−1^, which corresponds to the characteristic absorption peak of CCP alone. Meanwhile, the typical absorption peak of C-H near 2930 cm^−1^ from CCP was also present in the CCP_0.11_/CCHG and CCP_0.55_/CCHG hydrogels. In addition, the absorption peaks of CCP hydrogel groups around 1600 cm^−1^ and 1400 cm^−1^ were significantly enhanced compared with the CCHG hydrogel group, indicating uronic acid moieties were successfully introduced into composite hydrogels. Collectively, these results indicate the successful fabrication of CCHG composite hydrogels with varying CCP concentrations.

### 3.6. Rheological and Pharmacokinetic Properties of CCP Hydrogels

**Figure 6:**
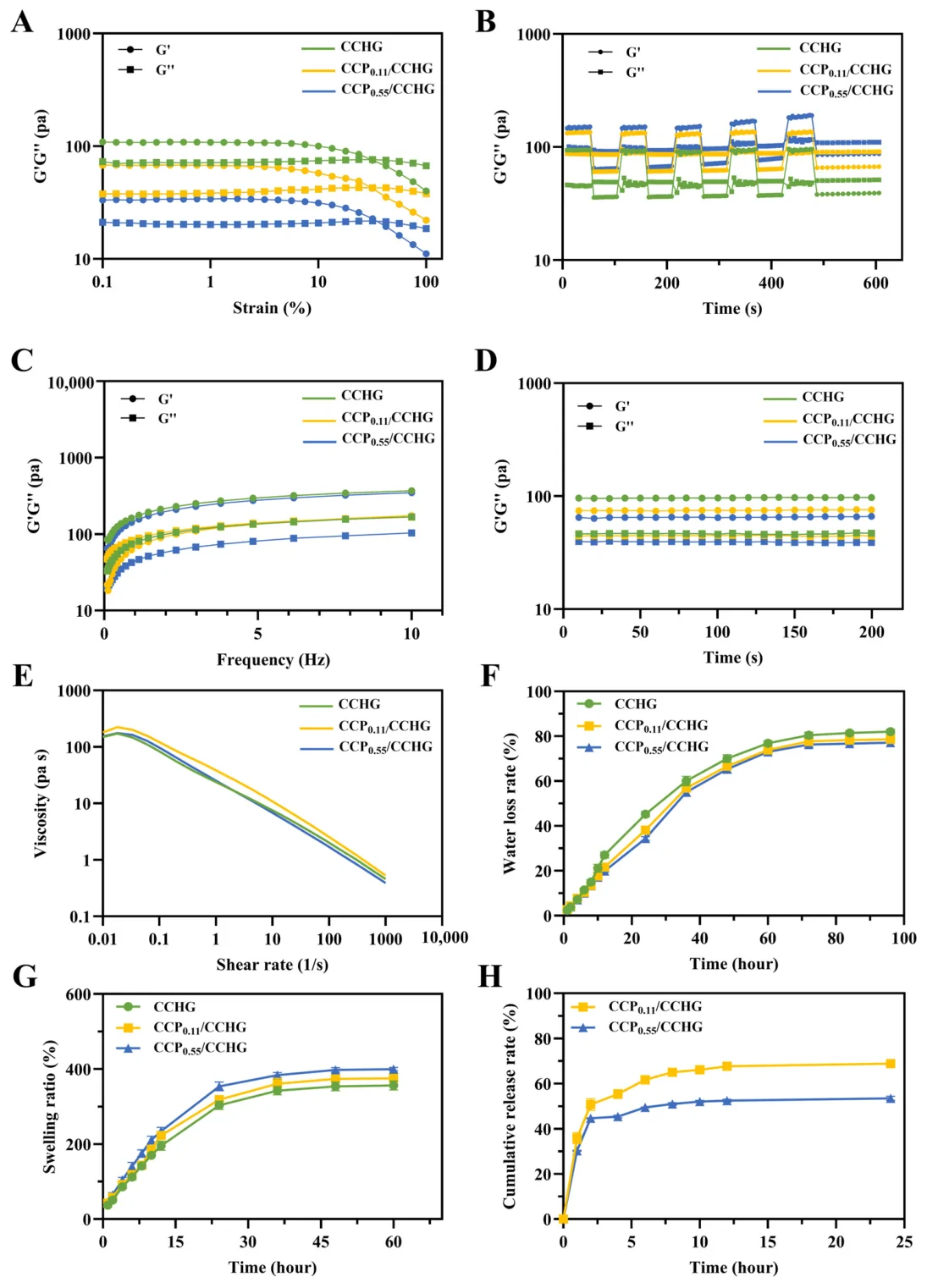
The physicochemical and rheological properties of hydrogels. (**A**) Strain sweep measurements, (**B**) alternating strain sweep measurements, (**C**) dynamic frequency sweep measurements, (**D**) dynamic time sweep measurements, (**E**) viscosity shear rate curves of CCHG, CCP_0.11_/CCHG, and CCP_0.55_/CCHG hydrogels. (**F**) Water loss rate curves of hydrogels at 37 °C. Time-dependent (**G**) swelling ratio and (**H**) cumulative CCP release profiles of hydrogels in PBS at 37 °C. Data are expressed as the standard error of the mean (*n* = 3).

The mechanical properties, self-healing capacity, and viscosity characteristics of hydrogels are crucial for their practical application as wound dressings [60]. Strain amplitude sweep analysis was first performed to investigate the critical strain value for breaking the hydrogel network. As shown in Figure 6A, the CCHG hydrogel maintained its elastic behavior (G′ > G″) until reaching a critical strain threshold (30.95%), beyond which the hydrogel network collapsed and underwent a sol–gel transition. Notably, with increasing CCP content, the critical strain thresholds of the composite hydrogels increased to 31.39% and 34.53%, respectively. This observation indicates that the incorporation of CCP could contribute to further strengthening the structural robustness of the hydrogel, enabling it to withstand greater external deformation without compromising its structural integrity. The self-healing capacity of hydrogels allows them to resist sustained physiological stresses, thereby extending their effective service life and supporting sustained therapeutic functions [61]. Continuous cyclic strain testing was further conducted to investigate the recoverability of the obtained hydrogels (Figure 6B). Our results reveal that hydrogels transitioned to a sol–gel state (G″ > G′) at 100% strain. When the strain was reduced to 5%, all three hydrogels rapidly reorganized and recovered their original structural integrity, indicating their excellent self-healing potential. The dynamic frequency–time scanning results reveal that the storage modulus (G′) was consistently higher than the loss modulus (G″) over the entire scan range, indicating that all hydrogel samples maintained stable elastic solid structures (Figure 6C,D). Furthermore, the steady-state flow test revealed that the viscosities of all three hydrogels were high at a low shear rate but decreased quickly with increasing shear rate (Figure 6E). This shear-thinning behavior is a desirable characteristic for wound dressings, as it allows the hydrogel to be easily spread and cover the wound surface [62]. The adhesion test verified that the as-prepared hydrogels achieve strong interfacial adhesion on various substrates such as glass, plastic, and medical gauze, demonstrating the high mucoadhesive property of CCP hydrogels (Figure S5). Overall, the rheological results demonstrate that CCP-loaded composite hydrogels possessed excellent stability, shear-thinning, and adherence properties, making them suitable for wound dressing applications.

Accumulating evidence has demonstrated that a moist microenvironment is more conducive to tissue regeneration and skin function restoration [63]. Hydrogels with high moisture retention capabilities are anticipated to exhibit superior efficacy in wound healing applications [64]. Our water retention assay results reveal that all three hydrogels exhibited a gradual increase in water loss rate over time (Figure 6F). Compared with the CCHG hydrogel, the CCP-loaded composite hydrogels showed higher water holding capacity. Their water retention rates reached 80% and 64% at 12 h and 24 h, respectively. Specifically, these two composite hydrogels still maintained a water retention rate of 22% after 96 h. Next, the swelling behaviors of the as-prepared hydrogels were investigated to evaluate their capacities for exudate absorption (Figure 6G). All three types of hydrogels exhibited rapid swelling within the initial 24 h, then they continued to swell slowly until 36 h and reached a plateau. Moreover, the drug release profiles of the hydrogels were also investigated (Figure 6H). Our results reveal that CCP was released from the obtained composite hydrogels at fast rates during the first 6 h, after which the rate gradually slowed down and reached a plateau after 12 h. The cumulative release rates of CCP from CCP_0.11_/CCHG and CCP_0.55_/CCHG were 52.65% and 69.09%, respectively. Therefore, the fabricated composite hydrogel enables efficient local release of CCP at the wound site, sustaining drug concentrations within the lesion region over time. Overall, CCP hydrogels have good rheological and viscosity characteristics, high water retention and hygroscopicity, and a stable drug release profile, making them ideal for administering to wounds.

### 3.7. The Antibacterial Activity of Hydrogels

Our previous results verify that CCP exhibited certain antibacterial activity. Given the critical role of antibacterial properties in dressings for treating DW, we further evaluated the in vitro antibacterial efficacy of CCP-loaded composite hydrogels. Different types of hydrogels were separately incubated with *E. coli* and *S. aureus*, and the bacterial colonies were imaged and counted after 24 h of incubation on agar plates. Our results reveal that the antibacterial activity of CCP/CCHG hydrogels was significantly enhanced with increased CCP loading as compared with the control group (Figure S6). These findings confirm that CCP/CCHG hydrogels preserve the antibacterial activity of CCP, which may contribute to overcoming infection-related delayed healing in DW.

### 3.8. Safety Evaluation of Hydrogels

Comprehensive safety evaluation of hydrogels is the most prerequisite for their further clinical application. The hemocompatibility of the prepared hydrogels was initially assessed using a hemolysis test, and the results are presented in Figure S7A. The positive control (1% Triton X-100) caused significant hemolysis of RBCs, resulting in the release of large amounts of hemoglobin into the supernatant. In contrast, the hemolytic rate of the three hydrogel groups was less than 5%, which was similar to that of the negative group (saline). These results demonstrate that the prepared hydrogels had no hemolytic effect on RBCs and showed high blood compatibility according to the ASTM standard F-756-08 [65].

As endothelial cells are critical components of the vascular system, their viability is essential for the integration of biomaterials with surrounding tissues. Therefore, the cytocompatibility of the obtained hydrogels was further evaluated using the CCK8 assay on HUVECs. Our results indicate that neither CCHG nor CCP-loaded composite hydrogels exerted significant toxic effects on HUVECs (Figure S7B). These results reveal that these hydrogels would not impair the function of endothelial cells or trigger an inflammatory response due to cell damage, which was particularly important for their potential applications in wound healing and tissue engineering.

In addition, a skin irritation test was conducted on rats to verify the safety of the hydrogels for topical administration. As expected, the tested hydrogels did not show any type of irritation (such as redness, swelling, or inflammation) on the rat skin at 24, 48, and 72 h after treatment with the indicated hydrogels (Figure S8A). Additionally, H&E staining of the skin tissues showed that the control and hydrogel treatment groups maintained normal morphology, with no evidence of inflammatory cells infiltrating or damaging the tissues (Figure S8B). Due to their non-irritant properties, CCP-loaded hydrogels can be safely applied to the skin surface without causing irritation to local tissues or allergic reactions, which is a significant factor in their development as topical dressings and skin repair materials.

### 3.9. In Vivo Wound Healing Assessment of Hydrogels

**Figure 7:**
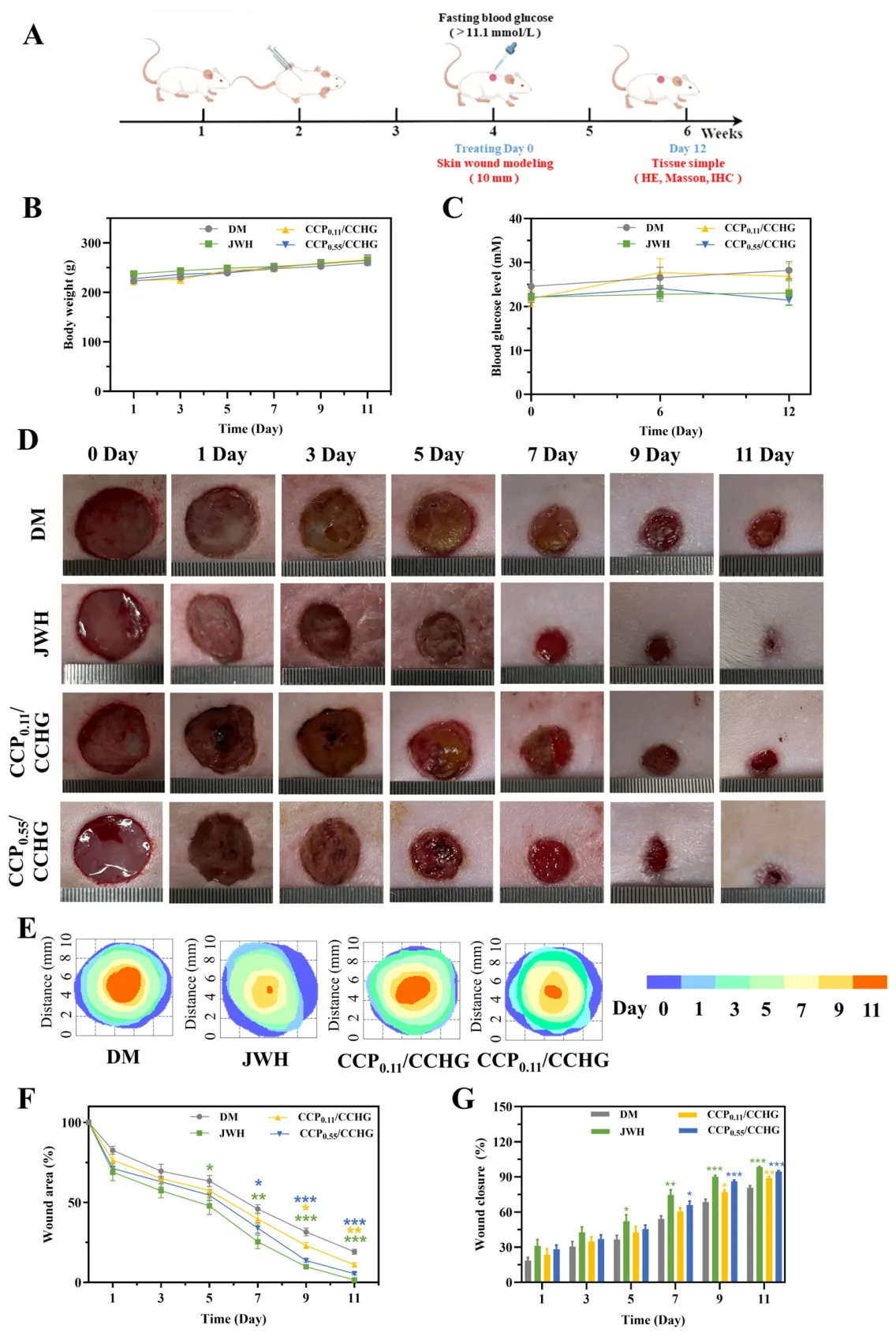
CCP hydrogels accelerated wound healing of full-thickness skin defects in diabetic rats. (**A**) Schematic illustration of the entire in vivo wound healing experiments. (**B**) Body weight changes in rats in each group during therapy. (**C**) Blood glucose level changes in the rats in each group during treatment. (**D**) Representative images of diabetic wound healing with different treatment groups on days 0, 1, 3, 5, 7, 9, and 11. (**E**) Schematic diagram of wound contraction at different time points during treatment. Quantitative analysis of (**F**) wound areas and (**G**) wound closure rate at different time points in each group. Data are expressed as standard error of the mean (*n* = 4). * *p* < 0.05, ** *p* < 0.01, *** *p* < 0.001 versus the DM group.

Next, a full-thickness dorsal wound model of diabetic rats was constructed to evaluate the pro-healing effect of CCP-loaded composite hydrogels on DW. Given its extensive clinical use for chronic wounds, such as DW in China, and its common application as a positive control for the in vivo wound healing assessment of natural polysaccharides, JWH was adopted as the positive control in this study [66]. The entire animal experimental process is schematically shown in a workflow diagram (Figure 7A). It was observed that body weights of the rats in all groups gradually increased throughout the experimental period (Figure 7B). Meanwhile, the blood glucose levels of all rats were significantly increased and maintained stable hyperglycemia during treatment, indicating that the model of diabetic rats was successfully established (Figure 7C). Following the establishment of the wound model, the wound area of rats in all groups gradually diminished over time, and the control group appreciably maintained a persistently slower wound healing rate relative to the treatment groups at all stages of the healing process (Figure 7D). The dynamic changes in wound area also revealed that both CCP-loaded composite hydrogels and positive drug JWH treatment in DW significantly enhanced wound healing (Figure 7E). In the later stage of wound healing, specifically on the 11th day, the wound healing rate of the rats in the CCP_0.55_/CCHG hydrogel group exceeded 94%, and its effect was near that of the JWH group (Figure 7F,G). Although the pro-healing effect of the CCP_0.11_/CCHG hydrogel was slightly lower than that in the CCP_0.55_/CCHG hydrogel group, its wound healing rate still reached 89% on day 11 post-treatment, which was significantly higher than the approximately 80% rate achieved by the same type of hydrogels. These results also confirm that the improvement in wound healing capacity was largely attributed to the CCP content of the composite hydrogels, where higher CCP content indicated higher wound healing efficacy.

**Figure 8:**
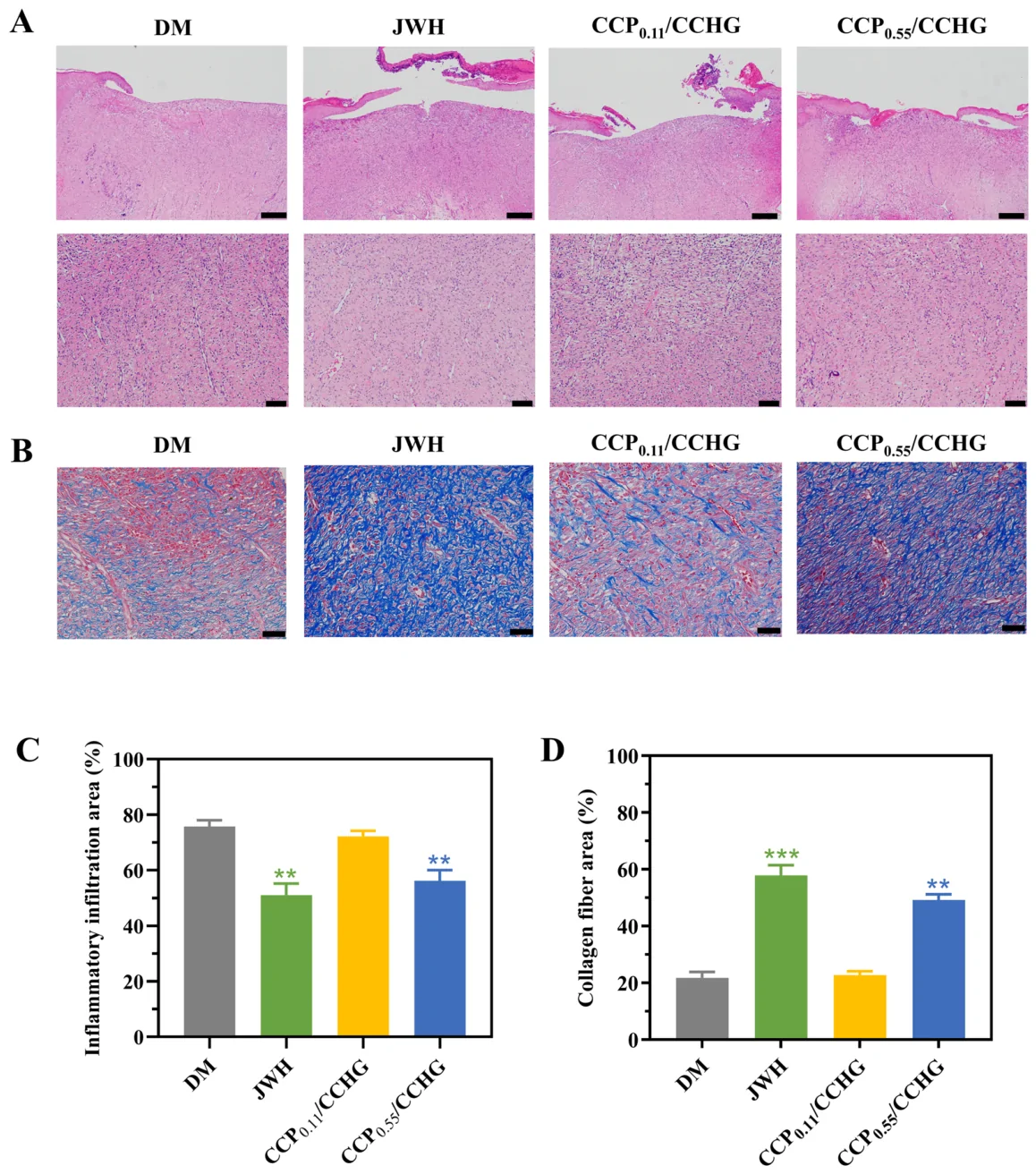
Histological evaluation of wound tissue sections. (**A**) Representative images of hematoxylin and eosin (H&E) staining of wound tissues (scale bar = 100 μM). Representative inflammatory infiltrate of H&E staining of diabetic wounds in each group at high magnification (scale bar = 50 μM). (**B**) Representative Masson’s trichrome staining images of wound tissues (scale bar = 25 μM). The corresponding quantitative data of (**C**) the inflammatory cell infiltration area and (**D**) collagen deposition. Data are expressed as the standard error of the mean (*n* = 3). ** *p* < 0.01, *** *p* < 0.001 versus the DM group.

Furthermore, H&E and Masson’s trichrome staining analyses were performed to comprehensively evaluate the extent of wound healing and regeneration. Compared with the control and CCP_0.11_/CCHG groups, treatment with CCP_0.55_/CCHG hydrogel exerted a more pronounced inhibitory effect on inflammatory cell infiltration and enhanced re-epithelialization, ultimately resulting in the formation of a more intact epidermal layer covering the wound surface (Figure 8A,C). Adequate and well-distributed collagen deposition is crucial for enhancing the tensile strength of the wound tissue and promoting the remodeling of the extracellular matrix, which are key characteristics of effective wound healing [67]. Compared with the control group, more uniform and denser collagen distributions were presented in the CCP_0.55_/CCHG and JWH treatment groups, which was beneficial for the wound healing process (Figure 8B). The quantitative analysis also confirmed that the collagen fiber level was elevated more than twofold in the rat skin tissues following CCP_0.55_/CCHG treatment compared with control tissues (Figure 8D).

Meanwhile, histological analysis of H&E-stained sections from the heart, liver, spleen, lungs, and kidneys of rats post-administration revealed no significant toxic lesions or structural abnormalities across all experimental groups, confirming that the CCP hydrogel exhibited no significant adverse effects (Figure S9).

**Figure 9:**
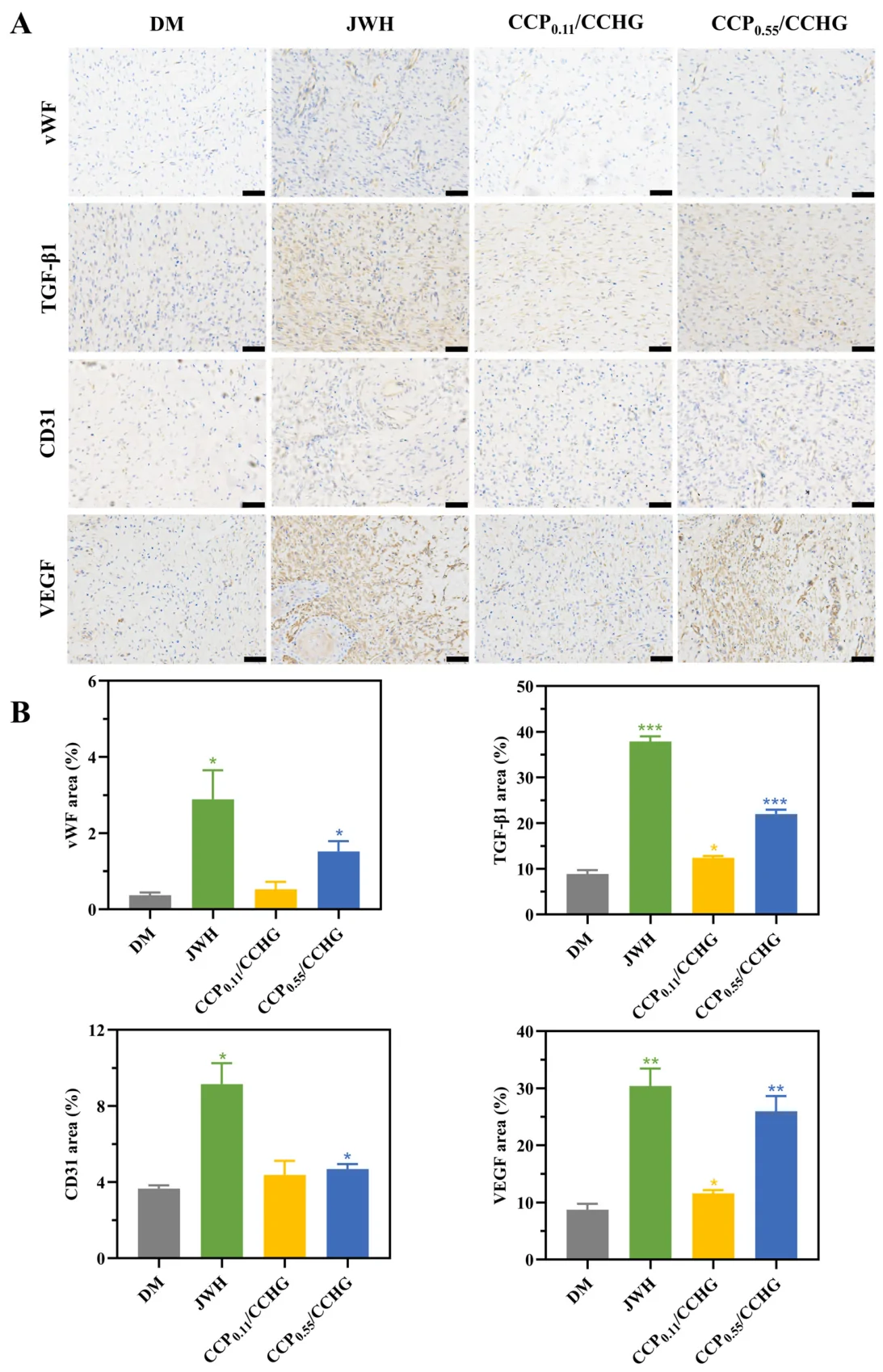
Immunohistochemical analysis of wound tissue from different treatment groups. Representative images of immunohistochemical staining of (**A**) vWF, TGF-β1, CD31, and VEGF (scale bar = 50 μM). Statistical graph of the expression of (**B**) vWF, TGF-β1, CD31, and VEGF. Data are expressed as the standard error of the mean (*n* = 3). * *p* < 0.05, ** *p* < 0.01, *** *p* < 0.001 versus the DM group.

To explore the underlying molecular mechanism by which CCP-loaded composite hydrogels promote DW healing, immunohistochemical staining was conducted to detect the expression of key proteins that govern the rates and quality of wound healing in skin tissues after different treatments. As a specific marker of vascular endothelial cells, vWF levels are positively correlated with the proliferation and migration of endothelial cells [68]. The expression of CD31 directly correlates with the density of newly formed blood vessels, as it is a key molecule for endothelial cell adhesion and tube formation [69]. TGF-β1 can stimulate angiogenesis directly or indirectly by upregulating the expression of other angiogenic factors, such as extracellular matrix (ECM) synthesis [70]. Moreover, vascular endothelial growth factor (VEGF) is a key upstream regulator of ECM remodeling in wound tissues, and stable VEGF expression is critical for fibroblast proliferation and migration, collagen and fibronectin deposition, and granulation tissue formation [71]. Compared with the control group, vWF, TGF-β1, CD31, and VEGF expression were significantly increased in the CCP_0.55_/CCHG treatment group (Figure 9A). Quantitative analysis demonstrated that an increase in CCP concentration within the hydrogel improved the expression of vWF, TGF-β1, and CD31 in a dose-dependent manner (Figure 9B). In addition, vWF and CD31 showed increased expression levels in the CCP_0.11_/CCHG treatment group, although the changes did not reach statistical significance compared with the control. Moreover, considering that excessive inflammation in DW leads to a pathological oxidative stress microenvironment that impairs re-epithelialization and delays wound healing, we also analyzed inflammation-related indicators for evaluating healing progression. As shown in Figure S10A, the CCP/CCHG and JWH treatment groups exhibited a notable reduction in brown-stained positive signals, indicating significantly downregulated expression levels of TNF-α and IL-6. In contrast, IL-10 expression was significantly elevated in these groups relative to the model group. Quantitative analyses further validated these observations (Figure S10B). These findings reveal that the hydrogel containing a high concentration of CCP effectively promoted key angiogenesis-related factors and VEGF, attenuated inflammation, thereby improving the wound tissue microcirculation and accelerating DW healing.

Taken together, in vivo experimental results confirm that the CCP hydrogel, especially the CCP_0.55_/CCHG formulation, efficiently accelerates DW healing. This beneficial effect is achieved by mitigating local inflammation, promoting collagen deposition, upregulating the expression of VEGF and key angiogenesis-related factors.

Notably, although our study validates the promising potential of CCP-loaded composite hydrogels for DW repair, several limitations should be acknowledged. Firstly, the fine structural features of CCP (such as the methylation, the composition of substituent groups, backbone linkages, and branching degree) remain uncharacterized. Clarifying these properties is essential for delineating CCP structure-activity relationships. Secondly, the HUVEC high-glucose and LPS-stimulated RAW264.7 models are common and useful, but they only capture selected aspects of diabetic wound pathology. Meanwhile, CCP hydrogels showed certain in vitro antibacterial activity, but infected wound animal models are necessary to further assess the anti-infective performance of CCP hydrogels in DW. Furthermore, while CCP hydrogels exhibit preliminary therapeutic effects on DW, their underlying molecular targets and mechanisms remain unclear. Although our study verified the preclinical wound healing potential of the CCP hydrogel in simplified DW models, further comprehensive validation is still essential to support its clinical translation.

## 4. Conclusions

In this study, a homogeneous yellow heteropolysaccharide with an average molecular weight of 39,697 Da, CCP, was isolated and purified from CC, and its effectiveness and underlying mechanism in the treatment of DW were investigated. In vitro experiments verified that CCP significantly enhanced the proliferation, migration, and tube formation of HUVECs in the HG environment. Meanwhile, CCP exhibited moderate antibacterial activity and could promote macrophage polarization from M1 to M2 phenotypes by downregulating pro-inflammatory factors in M1 macrophages and upregulating anti-inflammatory factors in M2 macrophages. Notably, CCP can form composite hydrogels with CBM940 and CMC-Na through physical crosslinking driven by multiple hydrogen bonds. The obtained composite hydrogels showed a porous, connected 3D network structure, which conferred them unique features, such as good rheological behaviors, long-sustained drug release, excellent moisturizing and swelling properties. In addition, these composite hydrogels preserve the antibacterial activity of CCP. In vivo results further demonstrate that these CCP-loaded composite hydrogels showed high biosafety and significantly promoted DW healing by alleviating the inflammatory microenvironment, improving angiogenesis, enhancing re-epithelialization, and collagen deposition. Overall, our results not only validate the feasibility of CCP as a potential bioactive component for wound repair but also highlight the considerable application prospects of CCP-loaded composite hydrogels as advanced wound dressings for DW healing.

## Figures and Tables

**Table 1:** Primer sequence.

Gene	Primer Sequence (5′-3′)
*GAPDH* forward	Forward: ACGGCCGCATCTTCTTGTGCA
*GAPDH* reverse	Reverse: ACGGCCAAATCCGTTCACACC
*IL-6* forward	Forward: TACCACTTCACAAGTCGGAGGC
*IL-6* reverse	Reverse: CTGCAAGTGCATCATCGTTGTTC
*IL-1β* forward	Forward: TGGACCTTCCAGGATGAGGACA
*IL-1β* reverse	Reverse: GTTCATCTCGGAGCCTGTAGTG
*TNF-α* forward	Forward: GGTGCCTATGTCTCAGCCTCTT
*TNF-α* reverse	Reverse: GCCATAGAACTGATGAGAGGGAG
*Arg-1* forward	Forward: CCCCAAAGGGATGAGAAGTT
*Arg-1* reverse	Reverse: GCTGAAGGTCTCTTCCATCACC
*IL-10* forward	Forward: CGGGAAGACAATAACTGCACCC
*IL-10* reverse	Reverse: CGGTTAGCAGTATGTTGTCCAGC
*TGF-β* forward	Forward: TGATACGCCTGAGTGGCTGTCT
*TGF-β* reverse	Reverse: CACAAGAGCAGTGAGCGCTGAA

**Table 2:** CCP hydrogels with different formulation composition.

Formulation	H_2_O (mL)	CCP (g)	CBM940 (mg)	CMC-Na (mg)
CCHG	11	0	100	125
CCP_0.11_/CCHG	11	0.11	100	125
CCP_0.55_/CCHG	11	0.55	100	125

**Table 3:** Elution profile of CCP.

RT (min)	lgMp	lgMw	lgMn	Mp	Mw	Mn	Area %
36.798	4.6	4.6	4.6	39,958	39,697	38,548	100

Mp, peak molecular weight; Mw, weight-average molecular weight; Mn, number-average molecular weight.

**Table 4:** HPLC chromatogram of derivatives of acid hydrolysates of CCP.

Name	Abbreviation	Mole Ratio	Content (μg/mg)
Glucose	Glc	0.474	117.76
Galacturonic acid	GalA	0.213	86.04
Arabinose	Ara	0.142	44.48
Galactose	Gal	0.108	40.36
Xylose	Xyl	0.020	6.30
Mannose	Man	0.020	7.40
Rhamnose	Rha	0.016	5.50

## Data Availability

The original contributions presented in this study are included in the article. Further inquiries can be directed to the corresponding authors.

## Supplementary Materials

The following supporting information can be downloaded at: https://www.mdpi.com/article/10.3390/biom16081162/s1. Figure S1: (**A**) A Standard curve for glucose by phenol-sulfuric acid methods. (**B**) Standard curve of glucuronic acid content; Figure S2: Calibration curve for molecular weight standards; Figure S3: Ion chromatogram of mixed 16 sugar; Figure S4: CCP decreased the level of p-STAT3 in HUVEC cells; Figure S5: Adhesion behavior of hydrogels on different materials; Figure S6: Antibacterial activity of hydrogels; Figure S7: The in vitro biocompatibility evaluation; Figure S8: Evaluation of skin irritation after application of hydrogels; Figure S9: Representative H&E staining images of major organs from rats treated with different formulations; Figure S10: Immunohistochemical analysis of wound tissue from different treatment groups.
