# Supplementary material for: Composite Hydrogel Loading Polysaccharides Derived from Coptis chinensis Franch. for Promoting Diabetic Wound Healing
Source: Biomolecules. 2026 Aug 10;16(8):1162. doi: 10.3390/biom16081162 (PMC13511239; doi:10.3390/biom16081162)
Supplement: Supplementary file 1 [file biomolecules-16-01162-s001.zip › biomolecules-4411252-supplementary.pdf]

Supporting Information

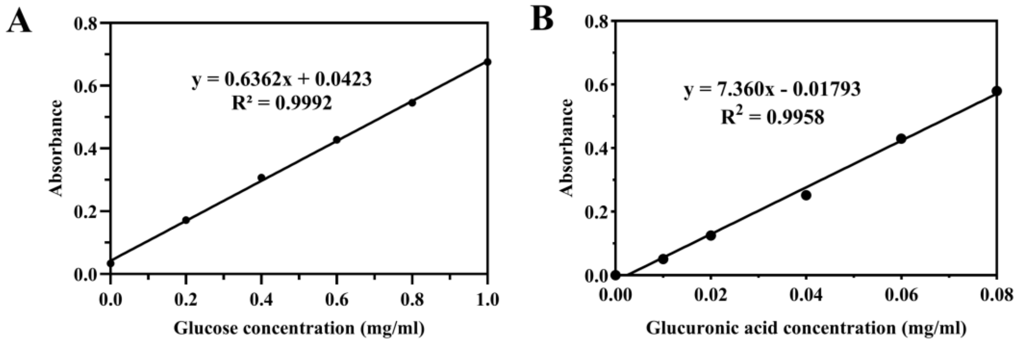

Figure S1. (A) A Standard curve for glucose by phenol-sulfuric acid methods. **(B)** Standard curve of glucuronic acid content.

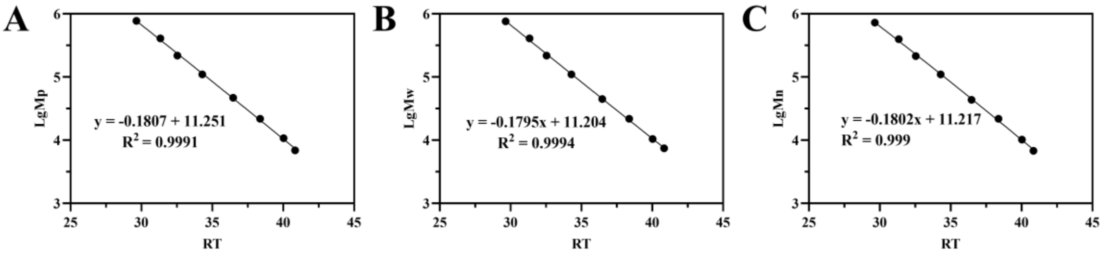

Figure S2. Calibration curve for molecular weight standards. **(A)** lgMp-RT (peak molecular weight - retention time). **(B)** lgMw-RT (weight average molecular weight - retention time). **(C)** lgMn-RT (number average molecular weight - retention time).

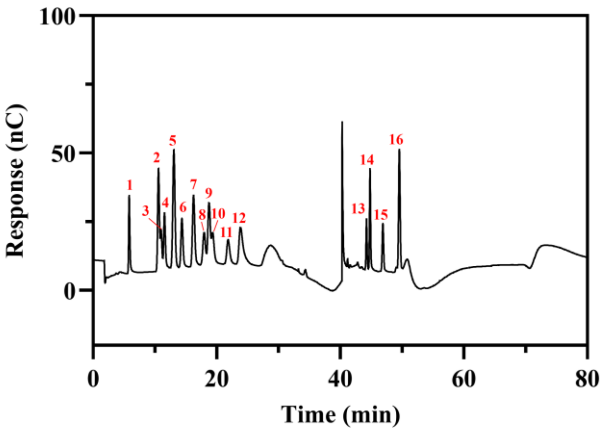

Figure S3. Ion chromatogram of mixed 16 sugar. 1. Fucose; 2. galactosamine hydrochloride; 3. rhamnose; 4. Arabinose; 5. glucosamine hydrochloride; 6. galactose; 7. glucose; 8. N-acetyl-D glucosamine; 9. xylose; 10. mannose; 11. fructose; 12. Ribose; 13. galacturonic acid; 14. guluronic acid; 15. glucuronic acid; 16. manuronic acid.

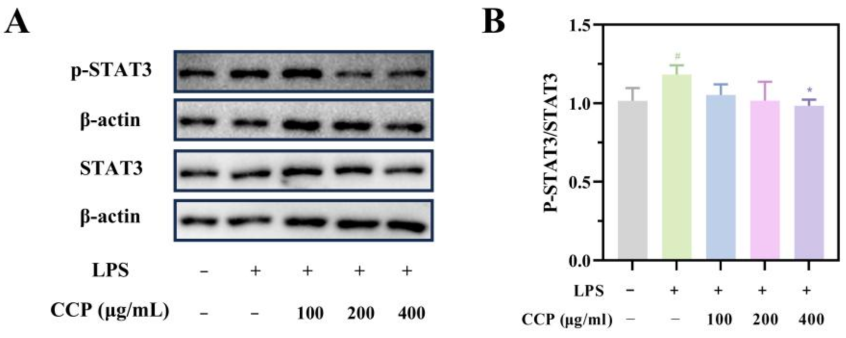

Figure S4. CCP decreased the level of p-STAT3 in HUVEC cells. (A) Western blot analysis of the expression of p-STAT3 and STAT3 in RAW cells were measured following treatment with LPS or LPS plus series concentrations of CCP. (B) The relative protein levels of p-STAT3 and STAT3 were quantitatively analyzed in each group. Data are expressed as standard error of the mean (*n* = 3). # $p < 0.05$ versus the control group. * $p < 0.05$ versus the LPS group.

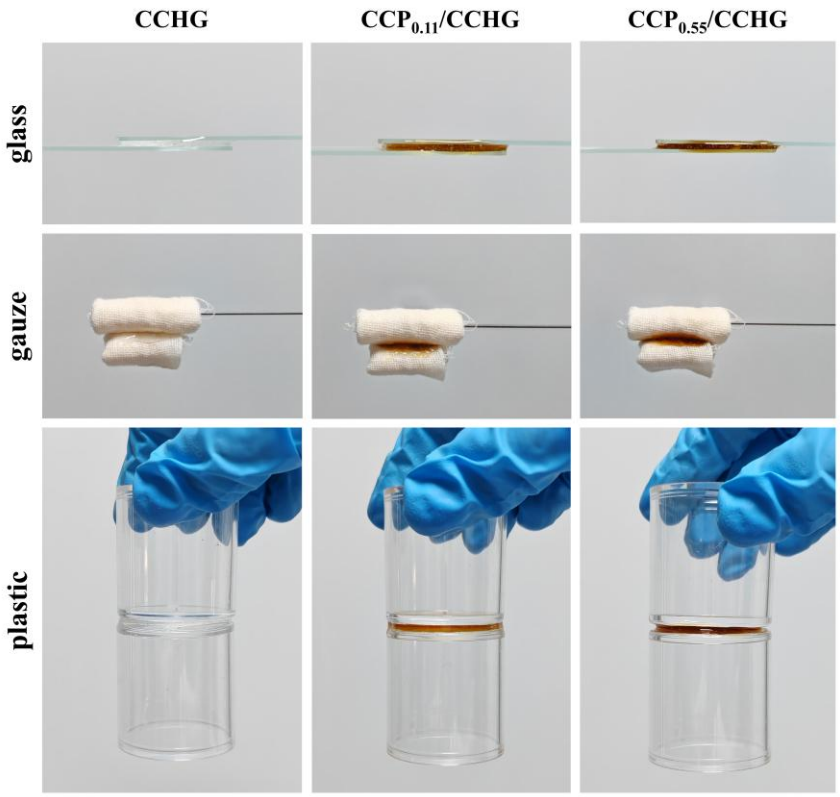

Figure S5. Adhesion behavior of hydrogels on different materials.

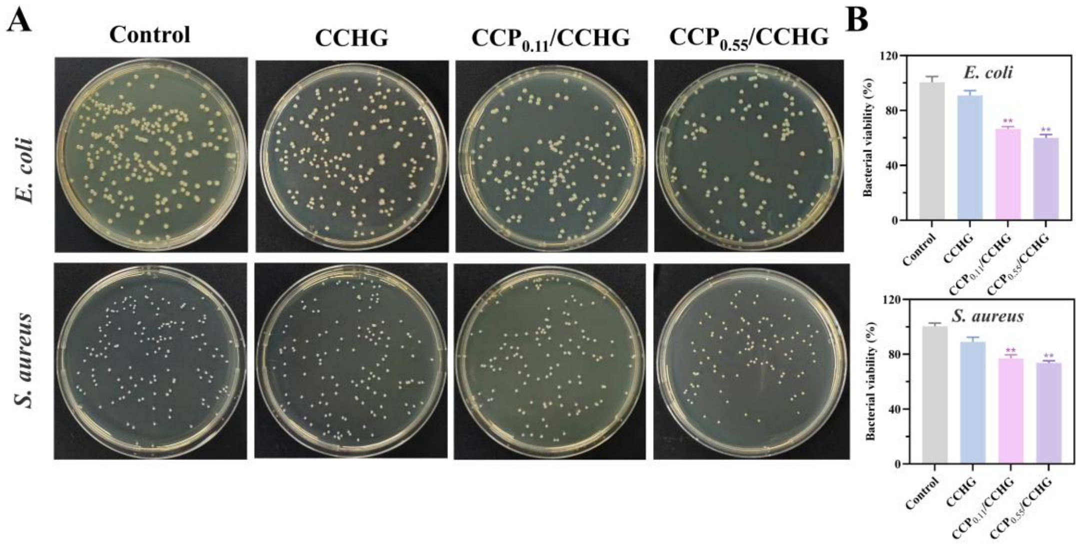

Figure S6. Antibacterial activity of hydrogels. (A) Photographs of plate count agar from different groups. (B) Corresponding antibacterial rate against *E. coli* and *S. aureus*. Data are expressed as standard error of the mean (n = 3). ** p < 0.01, *** p < 0.001 versus the control group.

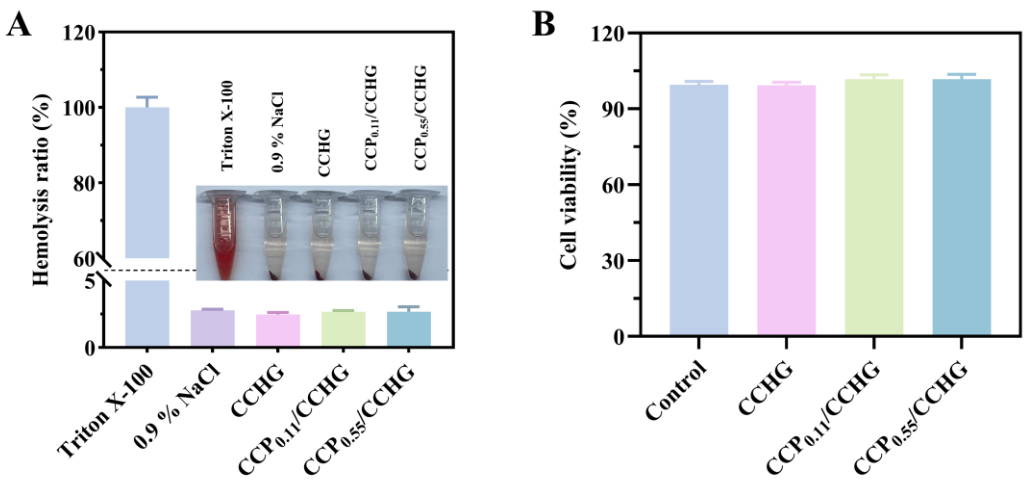

Figure S7. The in vitro biocompatibility evaluation. **(A)** Quantitative hemolysis ratio in different treatment groups, the insert graph is a photo of supernatants after red blood cells incubated with different solutions. Triton X-100 was set as the positive control and physiological saline was set as the negative control. **(B)** Relative viability of HUVECs estimated by CCK8 assay after 24 h incubation in different hydrogel extracts. Data were represented as Standard Error of the Mean (*n* = 3).

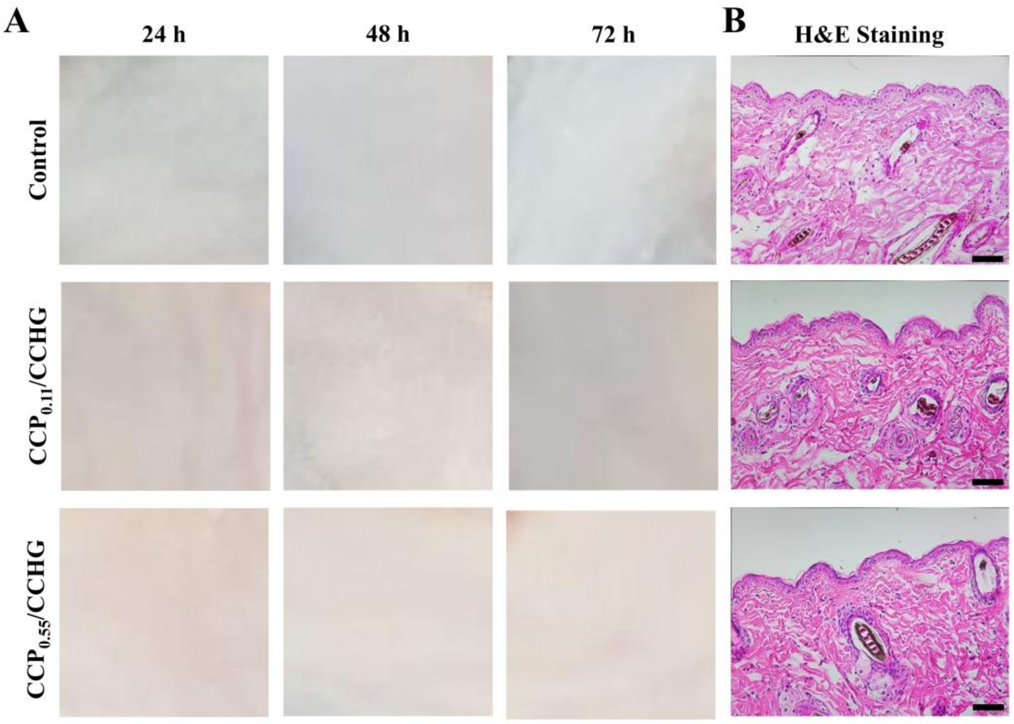

Figure S8**.** Evaluation of skin irritation after application of hydrogels. **(A)** Photographs of skin appearances and **(B)** H&E staining of dorsal skins (scale bar =50 μm).

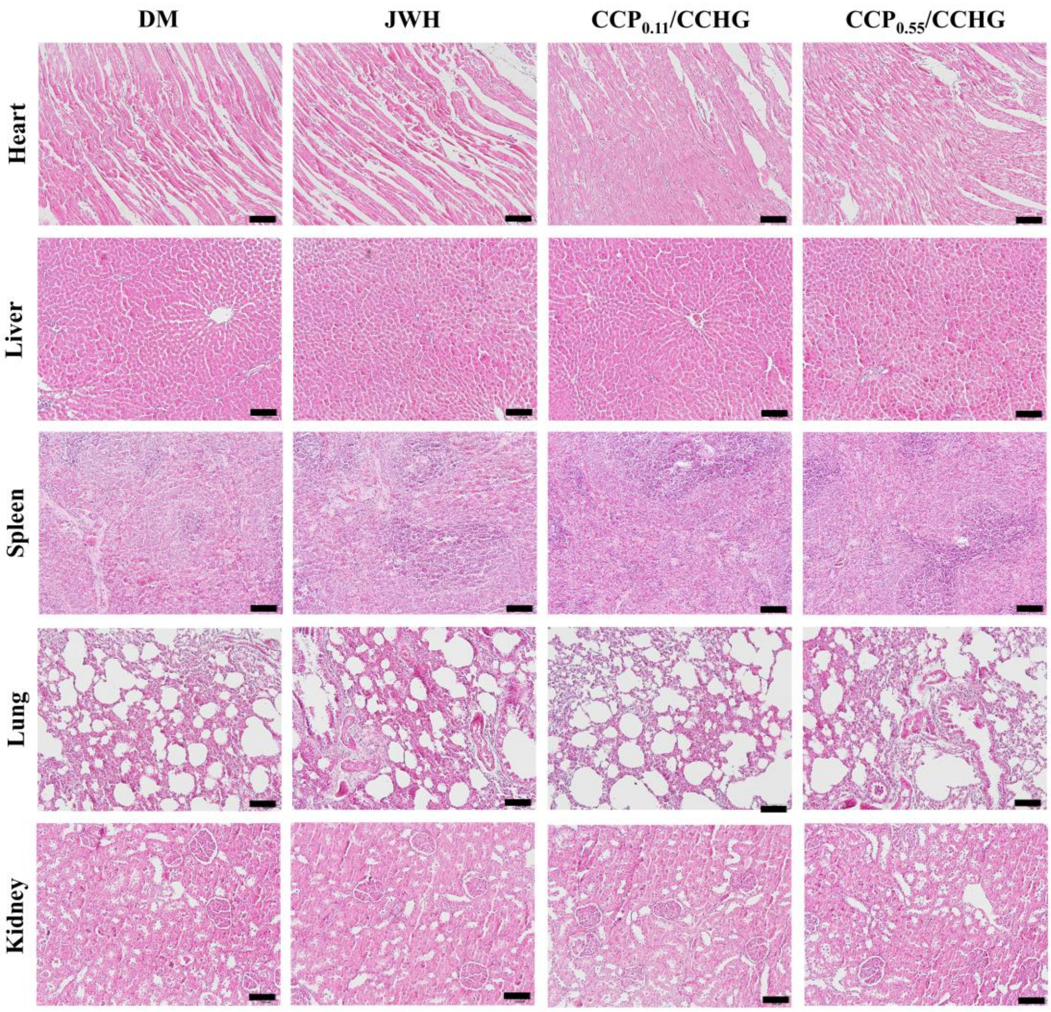

Figure S9. Representative H&E staining images of major organs from rats treated with different formulations (scale bar = 100 μm).

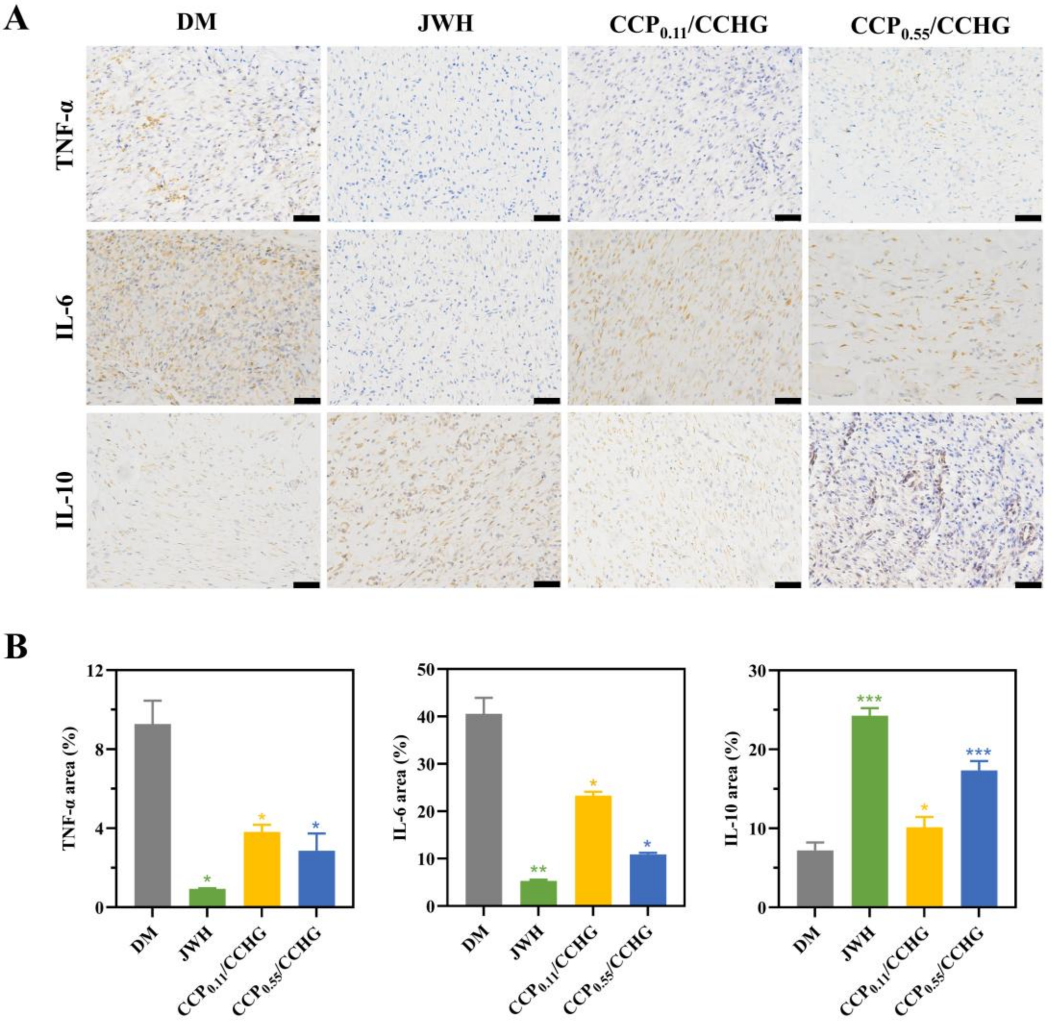

Figure S10**.** Immunohistochemical analysis of wound tissue from different treatment groups. Representative images of immunohistochemical staining of **(A)** TNF-α, IL-6 and IL-10 (Scare bar = 50 μM). The statistical graph on the expression of **(B)** TNF-α, IL-6 and IL-10. Data are expressed as standard error of the mean (n = 3). * $p < 0.05$, ** $p < 0.01$, *** $p < 0.001$ versus the DM group.
